# Simultaneous Clustering of Multiple Gene Expression and Physical Interaction Datasets

**DOI:** 10.1371/journal.pcbi.1000742

**Published:** 2010-04-15

**Authors:** Manikandan Narayanan, Adrian Vetta, Eric E. Schadt, Jun Zhu

**Affiliations:** 1 Department of Genetics, Rosetta Inpharmatics (Merck), Seattle, Washington, United States of America; 2 Department of Mathematics and Statistics, and School of Computer Science, McGill University, Montreal, Quebec, Canada; ETH Zurich, Switzerland

## Abstract

Many genome-wide datasets are routinely generated to study different aspects of biological systems, but integrating them to obtain a coherent view of the underlying biology remains a challenge. We propose simultaneous clustering of multiple networks as a framework to integrate large-scale datasets on the interactions among and activities of cellular components. Specifically, we develop an algorithm JointCluster that finds sets of genes that cluster well in multiple networks of interest, such as coexpression networks summarizing correlations among the expression profiles of genes and physical networks describing protein-protein and protein-DNA interactions among genes or gene-products. Our algorithm provides an efficient solution to a well-defined problem of jointly clustering networks, using techniques that permit certain theoretical guarantees on the quality of the detected clustering relative to the optimal clustering. These guarantees coupled with an effective scaling heuristic and the flexibility to handle multiple heterogeneous networks make our method JointCluster an advance over earlier approaches. Simulation results showed JointCluster to be more robust than alternate methods in recovering clusters implanted in networks with high false positive rates. In systematic evaluation of JointCluster and some earlier approaches for combined analysis of the yeast physical network and two gene expression datasets under glucose and ethanol growth conditions, JointCluster discovers clusters that are more consistently enriched for various reference classes capturing different aspects of yeast biology or yield better coverage of the analysed genes. These robust clusters, which are supported across multiple genomic datasets and diverse reference classes, agree with known biology of yeast under these growth conditions, elucidate the genetic control of coordinated transcription, and enable functional predictions for a number of uncharacterized genes.

## Author Summary

The generation of high-dimensional datasets in the biological sciences has become routine (protein interaction, gene expression, and DNA/RNA sequence data, to name a few), stretching our ability to derive novel biological insights from them, with even less effort focused on integrating these disparate datasets available in the public domain. Hence a most pressing problem in the life sciences today is the development of algorithms to combine large-scale data on different biological dimensions to maximize our understanding of living systems. We present an algorithm for simultaneously clustering multiple biological networks to identify coherent sets of genes (clusters) underlying cellular processes. The algorithm allows theoretical guarantees on the quality of the detected clusters relative to the optimal clusters that are computationally infeasible to find, and could be applied to coexpression, protein interaction, protein-DNA networks, and other network types. When combining multiple physical and gene expression based networks in yeast, the clusters we identify are consistently enriched for reference classes capturing diverse aspects of biology, yield good coverage of the analysed genes, and highlight novel members in well-studied cellular processes.

## Introduction

Heterogeneous genome-wide datasets provide different views of the biology of a cell, and their rapid accumulation demands integrative approaches that exploit the diversity of views. For instance, data on physical interactions such as interactions between two proteins (protein-protein), or regulatory interactions between a protein and a gene via binding to upstream regions of the gene (protein-DNA) inform how various molecules within a cell interact with each other to maintain and regulate the processes of a living cell. On the other hand, data on the abundances or expression of molecules such as proteins or transcripts of genes provide a snapshot of the state of a cell under a particular condition. These two data sources on physical interaction and molecular abundance provide complementary views, as the former captures the wiring diagram or static logic of the cell, and the latter the state of the cell at a timepoint in a condition-dependent, dynamic execution of this logic [1].

Researchers have fruitfully exploited this complementarity by studying the topological patterns of physical interaction among genes with expression profiles that are condition-specific [2], periodic [3], or correlated [4]; and similarity of the expression profiles of genes with regulatory, physical, or metabolic interactions among them [5]. Another line of research focuses on integrating the physical and expression datasets to chart out clusters or modules of genes involved in a specific cellular pathway. Methods were developed to search for physically interacting genes that have condition-specific expression (i.e., differential expression when comparing two or more conditions, as in “active subnetworks” [6]), or correlated expression (eg. subnetworks in the network of physical interactions that are coherently expressed in a given expression dataset [7]–[9]).

A challenge in expanding the scope of this research is to enable a flexible integration of any number of heterogeneous networks. The heterogeneity in the connectivity structures or edge density of networks could arise from the different data sources used to construct the networks. For instance, a network of coexpression relations between gene pairs is typically built using expression data of a population of samples (extracted from genetically varying individuals, or individuals subject to varying conditions/treatments). Whereas a network of physical interactions between protein or gene pairs is typically built by testing each interaction in a specific individual or *in-vitro* condition.

Towards addressing this challenge, we propose an efficient solution to a well-defined computational framework for combined analysis of multiple networks, each describing pairwise interactions or coexpression relationships among genes. The problem is to find common clusters of genes supported by all of the networks of interest, using quality measures that are normalized and comparable across heterogeneous networks. Our algorithm solves this problem using techniques that permit certain theoretical guarantees (approximation guarantees) on the quality of the output clustering relative to the optimal clustering. That is, we prove these guarantees to show that the clustering found by the algorithm on any set of networks reasonably approximates the optimal clustering, finding which is computationally intractable for large networks. Our approach is hence an advance over earlier approaches that either overlap clusters arising from separate clustering of each graph, or use the clustering structure of one arbitrarily chosen reference graph to explore the preserved clusters in other graphs (see references in survey [10]). JointCluster, an implementation of our algorithm, is more robust than the earlier approaches in recovering clusters implanted in simulated networks with high false positive rates. JointCluster enables integration of multiple expression datasets with one or more physical networks, and hence more flexible than other approaches that integrate a *single* coexpression or similarity network with a physical network [7]–[9], or multiple, possibly cross-species, expression datasets *without* a physical network [11]–[13].

JointCluster seeks clusters preserved in multiple networks so that the genes in such a cluster are more likely to participate in the same biological process. We find such coherent clusters by simultaneously clustering the expression data of several yeast segregants in two growth conditions [14] with a physical network of protein-protein and protein-DNA interactions. In systematic evaluation of clusters detected by different methods, JointCluster shows more consistent enrichment across reference classes reflecting various aspects of yeast biology, or yields clusters with better coverage of the analysed genes. The enriched clusters enable function predictions for uncharacterized genes, and highlight the genetic factors and physical interactions coordinating their transcription across growth conditions.

## Results

### JointCluster: A simultaneous clustering algorithm

To integrate the information in multiple physical interaction and gene expression datasets, we first represent each dataset as a network or graph whose nodes are the genes of interest and edges indicate relations between gene pairs such as physical interaction between genes or gene products in physical networks, or transcriptional correlation between genes in coexpression networks. Given multiple graphs defined over the same set of nodes, a *simultaneous clustering* is a clustering or partition of the nodes such that nodes within each set or *cluster* in the partition are well connected in each graph, and the total cost of *inter-cluster* edges (edges with endpoints in different clusters) is low. We use a normalized measure to define the connectedness of a cluster in a graph, and take the cost of a set of edges to be the ratio of their weight to the total edge weight in the graph. These normalized measures on clustering quality, described in detail in Methods, enable integration of heterogeneous graphs such as graphs with varying edge densities, and are beneficial over simpler formulations as described in detail in a previous study on clustering a single graph [15]. Our work extends the framework used in the single graph clustering study to jointly cluster multiple graphs, such that the information in all graphs is used throughout the algorithm.

The algorithm we designed, *JointCluster*, simultaneously clusters multiple graphs using techniques that permit theoretical guarantees on the quality of the output clustering relative to the optimal clustering. Since finding the optimal clustering is a computationally hard problem, we prove certain approximation guarantees that show how the cluster connectedness and inter-cluster edge cost measures of the clustering output by our algorithm are reasonably close to that of the optimal clustering (as formalized in Methods, Theorem 2). The basic algorithm, to which these guarantees apply, works with sparse cuts in graphs. A cut refers to a partition of nodes in a graph into two sets, and is called *sparse-enough* in a graph if the ratio of edges crossing the cut in the graph to the edges incident at the smaller side of the cut is smaller than a threshold specific to the graph. Graph-specific thresholds enable search for clusters that have varying connectedness in different graphs. The main steps in the basic JointCluster algorithm are: approximate the sparsest cut in each input graph using a spectral method, choose among them any cut that is *sparse-enough* in the corresponding graph yielding the cut, and recurse on the two node sets of the chosen cut, until well connected node sets with no sparse-enough cuts are obtained.

**Figure 1:**
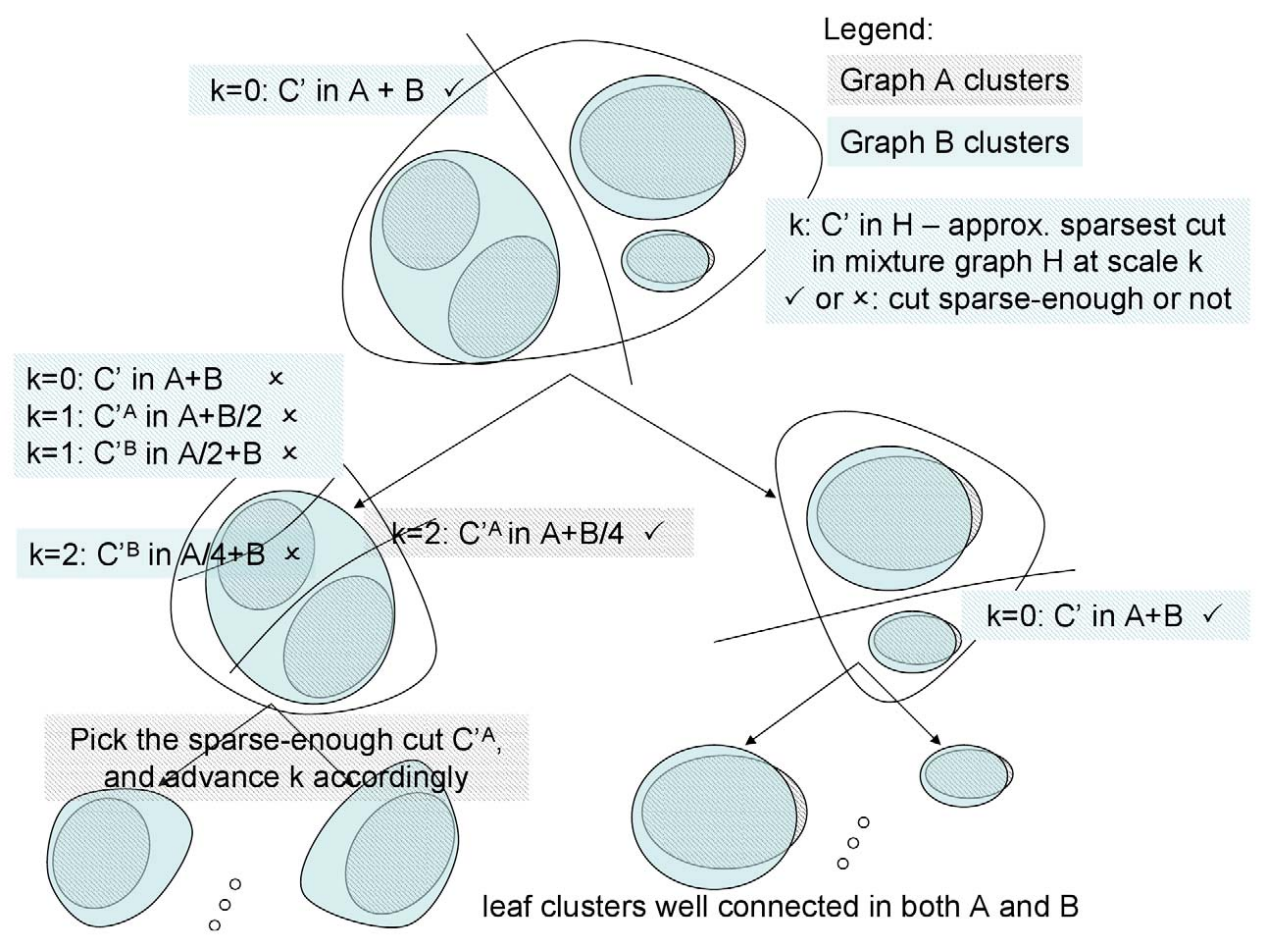
Schematic of JointCluster algorithm with scaling heuristic. Clustering tree produced from a simultaneous clustering of two networks 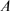 and 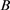. JointCluster can handle any number of networks, and the scaling heuristic transitions from a sum graph 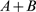 to the smallest of the sparse cuts in the individual graphs 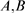 in increments of a scale parameter. At scale 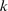, the graphs being analysed are 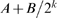 and 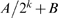. Only some steps of the scaling heuristic are shown here for simplicity, and complete description is provided in Methods. Graph-specific clusters are shown as different shaded regions within a larger set of nodes.

JointCluster implementation employs a novel scaling heuristic to reduce the inter-cluster edge cost even further in practice. Instead of finding sparsest cuts in input graphs separately as in the basic algorithm, the heuristic finds sparsest cuts in mixture graphs that are obtained from adding each input graph to a downscaled sum of the other input graphs. The mixture graph with unit downscaling is the sum graph whose edge weights are the sum of weights of the corresponding edges in all input graphs, and the mixture graphs with very large downscaling approaches the original input graphs. The heuristic starts with mixture graphs with small downscaling to help control inter-cluster edges lost in all graphs. But the resulting clusters are coarse (eg. clusters well connected in some graphs but split into smaller clusters in the rest are not resolved further). The heuristic then refines such coarse clusters at the expense of more inter-cluster edges by increasing the downscaling factor (see Figure 1). The scaling heuristic works best when combined with a cut selection heuristic: if for a particular downscaling, more than one mixture graph yields a sparse-enough cut, choose among them the cut that is sparse-enough in the most number of input graphs (breaking ties toward the cut with the least cost of edges crossing the cut in all graphs). A rigorous description of the algorithm with heuristics for advancing the downscaling factor and selection of cuts is provided in Methods.

Our method runs in an unsupervised fashion since algorithm parameters such as graph-specific thresholds are learnt automatically. The recursive cuts made by our algorithm naturally lead to a hierarchical clustering tree, which is then parsed objectively to produce the final clusters [16] using a modularity score function used in other biological contexts [17], [18]. The modularity score of a cluster in a graph is the fraction of edges contained within the cluster minus the fraction expected by chance in a randomized graph obtained from degree-preserved shuffling of the edges in the original graph, as described in detail in Supplementary Methods in Text S1. To aggregate the scores of a cluster across multiple graphs, we take their minimum and use this *min-modularity* score as the cluster score. The (min-modularity) score of a clustering is then the sum of the (min-modularity) scores of the constituent clusters.

### Benchmarking JointCluster on simulated data

We used simulated datasets to benchmark JointCluster against other alternatives: (a) 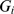 *Tree*: Choose one of the input graphs 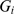 as a reference, cluster this single graph using an efficient spectral clustering method 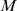 [16] to obtain a clustering tree, and parse this tree into clusters using the min-modularity score computed from all graphs; (b) *Coassociation*: Cluster each graph separately using the spectral method 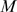, combine the resulting clusters from different graphs into a coassociation graph [19], and cluster this graph using the same method 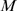. 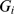 Tree method resembles the marginal cluster analysis in [20] as it analyses multiple networks using the clustering tree of a single network.

The simulated test data was generated as in an earlier study [18], under the assumption that the true classification of genes into clusters is known. Specifically, one random instance involved generating two test graphs 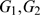 over 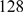 nodes each, and implanting in each graph the same “true” clustering of 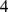 equal-sized clusters. A parameter 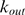 controlled the noise level in the simulated graphs by controlling the average number of inter-cluster edges incident at a node. The average number of total edges incident at a node was set at 16, so 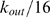 measures the false positive rate in a simulated graph. We used the standard Jaccard index, which ranges from 0 to 1, to measure the degree of overlap between the true clustering and the clustering detected by the methods. Please see Supplementary Text S1 for more details.

**Figure 2:**
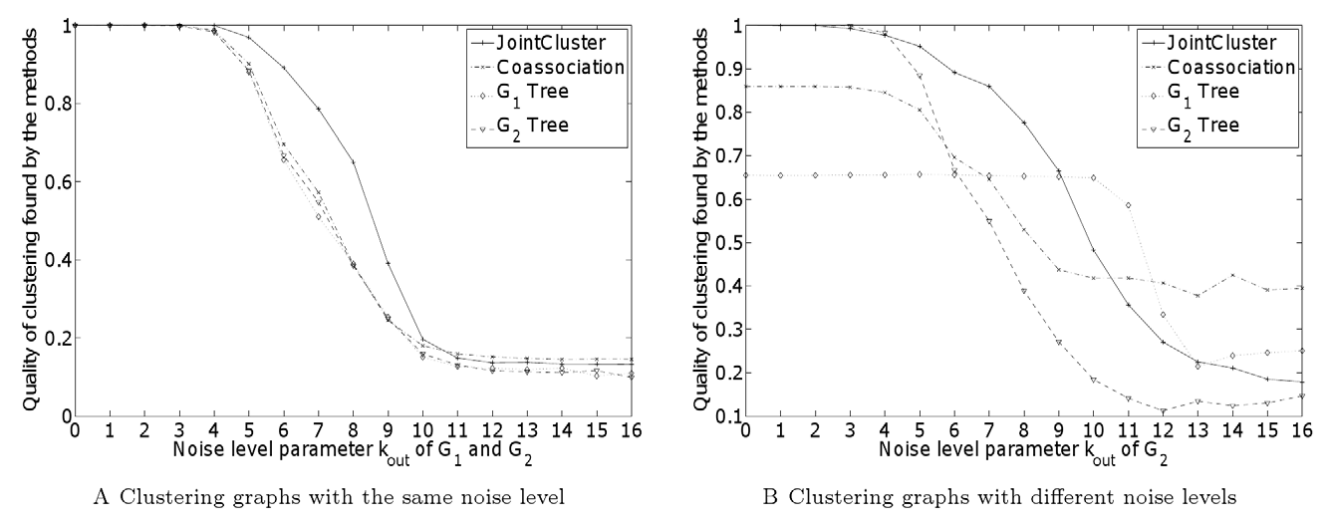
Benchmarking different clustering methods on simulated data. JointCluster detected implanted clusters on instances of randomly generated graphs 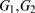 better than Coassociation and single tree methods, especially when the cluster structure was not strong, in two cases: (A) noise level in both 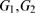 were varied together, (B) noise level 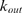 of 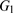 was fixed at 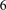 and of 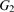 was varied from 0 to 16. The quality of the clustering detected by a method is measured as the standard Jaccard index measure between the detected and true clustering (y axis), averaged over all random instances for each setting of the noise level parameter 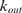 (x axis). The average number of edges incident at each node is 16, so 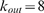 indicates a false positive rate of 50%.

Figure 2 A shows the performance of different methods in recovering common clusters in graphs 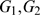 with the same noise level, averaged over 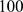 random instances of 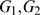 for each value of the noise level parameter 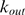. When the noise level is low (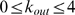 or false positive rate at most 25%), the clusters output by all methods are close to the true set of clusters (a Jaccard index close to 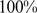). But when the noise level is high (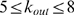 or false positive rate 25%–50%), the cluster structure becomes subtler, and JointCluster starts to outperform other methods and achieves the best improvement in Jaccard index over other methods at 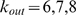. Note that values 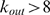 where false positive rates are above 50% do not lead to a meaningful cluster structure, and are only shown for context. Thus, within the setting of this benchmark, JointCluster outperformed the alternatives in recovering clusters, especially ones with a weak presence in multiple graphs.

To simulate real-world scenarios where the integrated networks could've different reliabilities, we benchmarked the methods on clustering graphs with different noise levels. Instead of varying the common 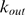 value of the 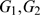 graphs as above, we fixed the noise level 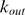 of 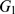 at 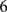 and varied the 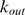 of the other graph 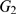 from 0 to 16. The relative performance of 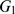 Tree and 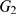 Tree methods (see Figure 2 B) showed that better clusters were obtained when clustering tree of the graph with the lower noise level was used. JointCluster integrated the information in the two graphs to produce a joint clustering tree, which when parsed yielded better clusters than Coassociation and single tree clusters for a larger range of the parameter values (see Figure 2 B). The empirical evaluation of JointCluster and competing methods was done using large-scale yeast datasets, and described in detail next.

### Systematic evaluation of the methods using diverse reference classes in yeast

Expression of 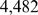 transcripts were measured in 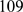 segregants derived from a cross between the BY and RM strains of the yeast *Saccharomyces cerevisiae* (denoted here as the BxR cross), grown under two conditions where glucose or ethanol was the predominant carbon source, by an earlier study [14]. From these expression data, we derived glucose and ethanol coexpression networks using all 4,482 profiled genes as nodes, and taking the weight of an undirected edge between two genes as the *absolute* value of the Pearson's correlation coefficient between their expression profiles. The network of physical interactions (protein-protein indicating physical interaction between proteins and protein-DNA indicating regulatory interaction between a protein and the upstream region of a gene to which it binds) among the same genes or their protein products, collected from various interaction databases (eg. BioGRID [21]), was obtained from an earlier study [9]. The physical network was treated as an undirected graph after dropping interaction orientations, and contained 41,660 non-redundant interactions.

We applied JointCluster and other clustering methods to integrate the yeast physical and glucose/ethanol coexpression networks, and assessed the biological significance of the detected clusters using reference sets of genes collected from various published sources. The reference sources fall into five diverse classes:

- *GO Process*: Genes in each reference set in this class are annotated to the same GO Biological Process term [22],
- *TF (Transcription Factor) Perturbations*: Genes in each set have altered expression when a TF is deleted [23] or overexpressed [24],
- *Compendium of Perturbations*: Genes in each set have altered expression under deletions of specific genes, or chemical perturbations [25],
- *TF Binding Sites*: Genes in a set have binding sites of the same TF in their upstream genomic regions, with sites predicted using ChIP binding data [26], [27], and
- *eQTL Hotspots*: Certain genomic regions exhibit a significant excess of linkages of expression traits to genotypic variations. Genes with expression linkages to such an eQTL (expression Quantitative Trait Loci) hotspot region are grouped into a reference set [14].

We overlapped the detected clusters with the reference sets in these classes to differentiate clusters arising from spurious associations from those with genes coherently involved in a specific biological process, or coregulated due to the effect of a single gene, TF, or genetic factors. The results are summarized using standard performance measures, sensitivity (fraction of reference sets significantly enriched for genes of some cluster output by a method) and specificity (fraction of clusters significantly enriched for genes of some reference set), both reported as percentages for each reference class. The significance cutoff for the enrichment P-value (denoted 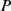 hereafter) is 0.005, after Bonferroni correction for the number of sets tested. The sensitivity measures the “coverage” of different biological processes by the clusters, and the specificity the “accuracy” of the clusters. We compared JointCluster with Coassociation [19], single graph [16], and single tree (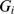 Tree) methods, and when applicable with competing methods, Matisse [9] and Co-clustering [7], which integrate a single coexpression network with a physical network. All reported results focus only on clusters with at least 10 genes.

**Figure 3:**
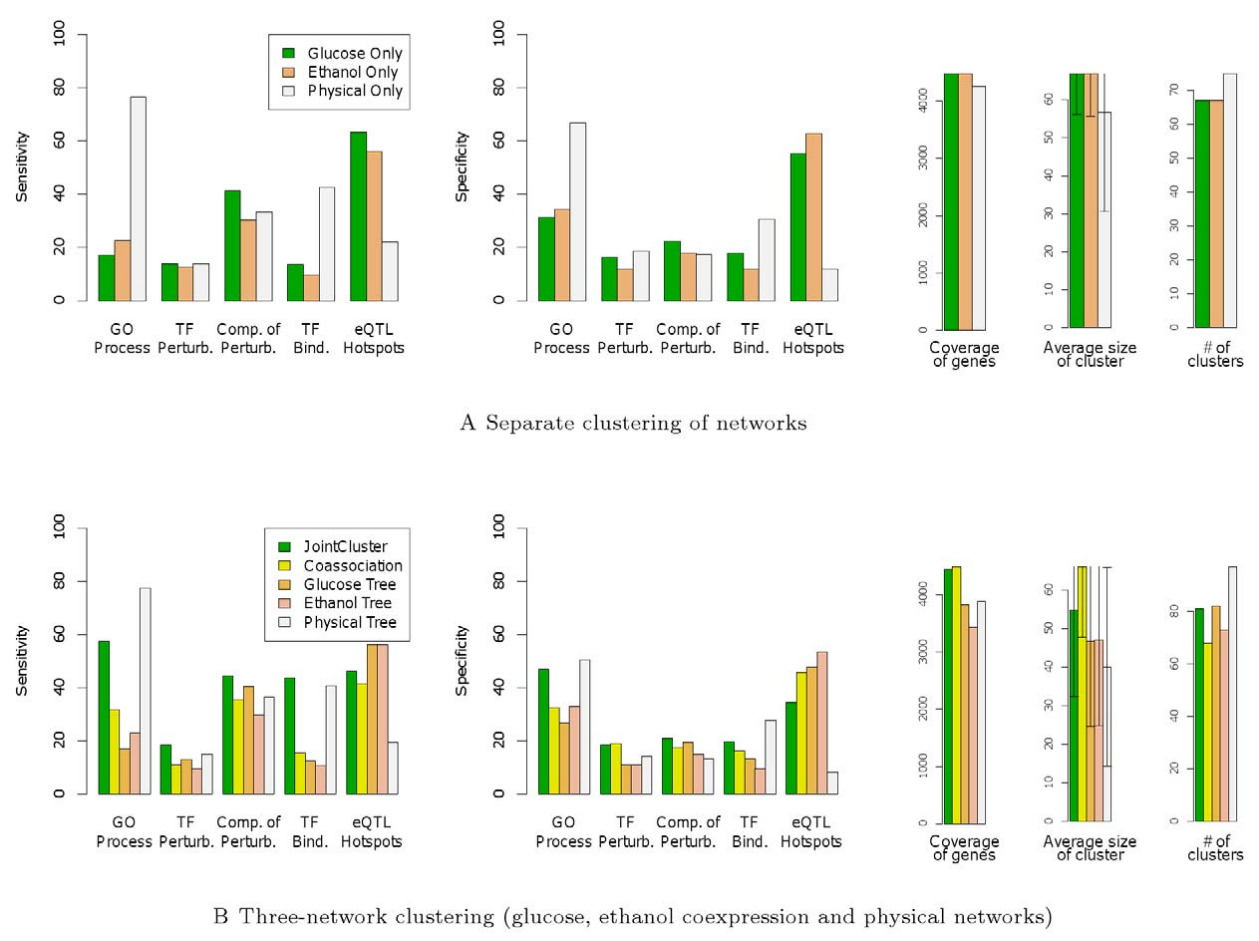
Sensitivity and specificity of clusters detected from the yeast networks. Performance of (A) single graph methods, and (B) JointCluster, Coassociation and single tree methods are shown. To help interpret these performance measures properly, information such as coverage of genes (# of genes in detected clusters) and average size of a cluster (average # of genes in a cluster with error bars indicating standard deviation) are also shown. The number of reference sets in GO Process, TF Perturbations (TF Perturb.), Compendium of Perturbations (Comp. of Perturb.), TF Binding Sites (TF Bind.) and eQTL Hotspots are 379, 315, 198, 103 and 41 respectively.

To provide context, we present results from clustering each network separately using the single graph method (Glucose/Ethanol/Physical Only) in Figure 3 A. Physical Only performs better than the other two methods *wrt* (with respect to) GO Process and TF Binding Sites, and Glucose/Ethanol Only fare well wrt eQTL Hotspots. This relative performance is not surprising due to the varying levels of bias in the reference classes, and the different data sources used to construct the networks. Though physical interactions between genes or gene products are known to be predictive of shared GO annotations, certain GO annotations inferred from physical interactions introduce bias. The same ChIP binding data [26] was used to predict TF binding sites and protein-DNA interactions, so validation of clusters derived from the physical network using TF Binding Sites is biased. Finally, the same expression data underlying the coexpression networks was used with the independent genotype data to define the eQTL hotspots [14]. Hence the eQTL Hotspots class does not by itself provide a convincing validation of the coexpression clusters; however it can be used to understand the extent of genetic control of coordinated transcription and to validate clusters derived from networks comprising only physical interactions. The reference classes offering truly independent validation of clusters are TF Perturbations and Compendium of Perturbations, and the three single graph methods perform similarly in these perturbation classes.

Integration of the yeast physical network with the glucose/ethanol coexpression networks was done to find sets of genes that clustered reasonably well in all three networks. JointCluster performed a better integration of these networks than Coassociation for all reference classes except eQTL Hotspots (Figure 3 B). The enrichment results of single tree methods in Figure 3 B followed a trend similar to the single graph methods in Figure 3 A, reflecting the bias in the reference classes. In the two truly independent perturbation classes, JointCluster showed better sensitivity than the other methods at comparable or better specificity.

In summary, though different single graph and single tree methods were best performers in different reference classes (from Figures 3 A and 3 B), JointCluster was more robust and performed well across all reference classes characterizing diverse cellular processes in yeast (Figure 3 B, first bar). The clusters identified by JointCluster that were consistently enriched for different reference classes are explored in depth next.

### Clusters preserved in the physical and coexpression networks are consistent with known biology

The clusters in a clustering were ordered by their min-modularity scores, and identified by their rank in this ordering. We highlight the biology and multi-network connectivity of the top-ranked clusters detected by JointCluster in an integrated analysis of the yeast physical and glucose/ethanol coexpression networks. The member genes and enrichment results of all preserved clusters detected by JointCluster are provided as Supplementary Data in Text S1 (see also Table 1 in Supplementary Text S1 for GO Process enrichment of many top-ranked clusters).

The preserved cluster with the best min-modularity score, Cluster #1, comprised 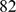 genes with a min-modularity score of 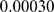. The respective modularity scores in the physical, glucose, and ethanol networks were 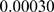, 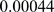, and 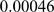, which were significantly higher than the modularity of a random set of genes of the same size in the respective networks (see Figure 1 in Supplementary Text S1 for the cluster's connectivity in the three networks). This cluster was significantly enriched for genes involved in the GO Processes, translation (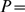
1e-20; see Table 1 in Supplementary Text S1 ), mitochondrion organization (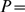
1e-20), mitochondrial translation (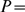
1.8e-17) and cellular respiration (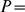
3.1e-8).

The enrichments noted for Cluster #1 is consistent with and even extend published results on this dataset. The shift in growth conditions from glucose to ethanol triggers large changes in the transcriptional and metabolic states of yeast [28], with the primary state being fermentation in glucose and respiration in ethanol. The transcription of functionally related genes, measured across different timepoints during the shift, are highly coordinated [28]. The coregulation of related genes is also evident from the clusters of coexpressed genes found under the glucose condition, using expression profiles of genetically perturbed yeast segregants from the BxR cross [29]. Our results take this evidence a step further, because the coexpression of cluster genes are elucidated by genetic perturbations in both growth conditions (regardless of the expression level changes of cluster genes between the conditions). We also note that the top-ranked cluster is significantly enriched for genes linking to the eQTL hotspot region glu11 in Chromosome 14 [14] (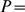
4.6e-25), which highlights the role of genetic factors in the coregulation of genes involved in (mitochondrial) translation and cellular respiration.

A different perspective on yeast biology in the glucose medium is offered by Cluster #2 consisting of 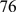 genes (with a significant min-modularity score 0.00021; see Figure 1 in Supplementary Text S1 ). This cluster is significantly enriched for ribosome biogenesis (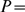
2.4e-37; see Table 1 in Supplementary Text S1 ), and related GO Process terms such as ribonucleoprotein complex biogenesis and assembly (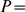
9.4e-37), ribosomal large subunit biogenesis (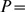
8.8e-35), and rRNA processing (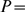
3.8e-33). Genes in this cluster significantly overlap with the perturbation signature of BUD21, a component of small ribosomal subunit (SSU) processosome (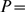
4.1e-15), and with genes whose expression links to genetic variations in the eQTL hotspot region glu12 in Chromosome 15 [14] (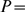
7.9e-16). These results are consistent with the literature on the regulation of yeast growth rate in the glucose or ethanol medium, achieved by coregulation of genes involved in ribosome biogenesis and subsequent protein synthetic processes [28].

To further understand the biological significance of these preserved clusters in physical and coexpression networks, we used the reference yeast protein complexes in MIPS [30] (comprising 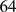 literature-based, small-scale complexes of at least five genes, at level at most two in the MIPS hierarchy). The enrichment of the joint clusters wrt this MIPS Complex class was 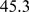
% sensitivity and 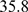
% specificity. Of the clusters not enriched for any MIPS complex, some were significantly enriched for other functionally coherent pathways (eg. Cluster #13 was enriched for amino acid biosynthetic process; see Table 1 in Supplementary Text S1 ). So the clusters detected by JointCluster overlapped with several known complexes or other functional pathways.

### JointCluster identifies subtle clusters

One of the goals of jointly clustering multiple networks is to identify subtle clusters: sets of genes that cluster reasonably well, but not strongly, in all networks. We start with biologically significant clusters i.e., clusters enriched for some reference set wrt *all* five reference classes, and test if any such cluster has a weak modularity score in some graph. We identified 5 biologically significant clusters using JointCluster: Clusters #4, #13, #15, #19, and #28. Table 2 in Supplementary Text S1 shows the reference sets they were enriched for, and Figure 2 in Supplementary Text S1 the modularity scores of Clusters #4 and #28.

Cluster #28, the biologically significant cluster with the lowest min-modularity score, had 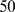 genes and was enriched for the GO Processes, multi-organism process (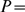
2.5e-12) and conjugation (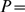
2e-10). This cluster's role in mating was further supported by its significant enrichment for perturbation signatures of STE12 (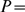
5.7e-9) and FUS3/KSS1 (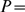
8.1e-21), because Ste12p is a TF regulating the expression of mating genes and is activated by the Fus3p/Kss1p kinases in the well-studied mitogen-activated protein kinase (MAPK) cascade [31]. Such a cluster of well-studied genes was recovered just by the single graph method Physical Only, but *not* by Glucose/Ethanol Only. Here we considered a cluster of genes to be *recovered* by a method if this cluster is significantly enriched for some cluster found by the given method (as in reference set enrichment). JointCluster was able to detect this cluster due to its high modularity in the physical network combined with its significant, albeit weak, modularity in the coexpression networks (see Figure 2 in Supplementary Text S1 ).

To explore more subtle clusters, we focused on the clusters identified by JointCluster that were enriched for at least four reference classes, instead of all five required above. Clusters #52 and #54 had the two lowest min-modularity scores among such clusters, and were each recovered just by the Physical Only method, but not by Glucose/Ethanol Only. Cluster #52 comprised of 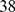 genes had a significant min-modularity score (see Figure 3 in Supplementary Text S1 ), and was enriched for the GO Process, ubiquitin-dependent protein catabolic process (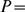
4.4e-23). RPN4 is a TF involved in regulation of the protein catabolic process [32], and this cluster was significantly enriched for genes in the deletion signature of RPN4 (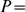
1.4e-9) and genes with predicted binding sites of RPN4 (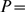
1.8e-23; see Supplementary Data in Text S1 for other enrichments). These examples reiterate how a combined analysis of multiple networks by JointCluster detects meaningful clusters that would be missed by separate clustering of the networks.

### Preserved clusters inform on uncharacterized ORFs

Despite the intense focus on elucidating yeast biology by many researchers, roughly 1,000 Open Reading Frames (ORFs) are still uncharacterized [33]. Therefore, predicting the function of these ORFs is important to guide future experiments towards strains and perturbations that likely elucidate these ORFs [33]. While there have been many network-based function prediction studies (see survey [34]), our study provides a different perspective by using clusters preserved across *multiple* coexpression and physical networks. Our prediction strategy, based on a module-assisted guilt-by-association concept [34], annotates the uncharacterized ORFs in a cluster detected by JointCluster to the GO Process reference set for which this cluster is most significantly enriched.

To test the utility of these predictions for a well-studied process in yeast, we focused on clusters enriched for ribosome biogenesis (Clusters #2 and #22; see Table 1 in Supplementary Text S1 ). Two ORFs in Cluster #2, a top-ranked cluster discussed above, were marked as uncharacterized by SGD [35] (April 2009 version): YER067W and YLR455W. Our predictions for these ORFs have different types of support: YER067W is significantly correlated with 67 and 33 of the 76 genes in this cluster in glucose/ethanol expression datasets respectively (Pearson's correlation test 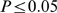, Bonferroni corrected for the cluster size), and YLR455W has known protein-protein interactions with five other genes in the cluster, NOC2, BRX1, PWP1, RRS1, EBP2, all of which were implicated in ribosome biogenesis. Cluster #22 had 9 uncharacterized ORFs, YIL096C, YOR021C, YIL091C, YBR269C, YCR087C-A, YDL199C, YKL171W, YMR148W, and YOR006C. Two of them (YIL096C and YOR021C) have predicted roles in ribosome biogenesis based on function predictions collected from the literature by SGD for some of the uncharacterized ORFs. This lends support to the two predictions and leaves the other novel predictions for further validation. All of the uncharacterized ORFs in Cluster #22 except YBR269C were significantly correlated with more than three-fourths of the 35 genes in the cluster in both glucose/ethanol expression datasets (using the same criteria above based on Pearson's correlation test). The predictions here were based on either support from the physical network (for YLR455W) or from both coexpression networks (for the rest), and hence illustrates the advantage of using multiple data sources.

Of the 990 ORFs classified as uncharacterized by SGD (April 2009 version), 524 overlapped with the genes used to build the yeast networks. We could predict the function for 194 of them, by virtue of their membership in preserved clusters significantly enriched for some GO Process term. Using single graph (Glucose/Ethanol/Physical Only) clusters in place of the preserved clusters detected by JointCluster yielded predictions for 143, 148 and 247 uncharacterized ORFs respectively, reflecting the relative GO Process specificity of these methods (Figures 3 A and 3 B). The relative number of predictions from different methods should be viewed in context of the systematic evaluations above, which showed that whereas Physical Only performed best wrt GO Process, JointCluster produced clusters that were more coherent across all reference classes. The predictions from JointCluster were also complementary to those from Physical Only, with the functions of only 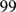 uncharacterized ORFs predicted by both methods. The functions predicted using the preserved clusters are available as Supplementary Data in Text S1 , and point to well-studied biological processes that have escaped complete characterization.

### JointCluster yields better coverage of genes than a competing method

**Figure 4:**
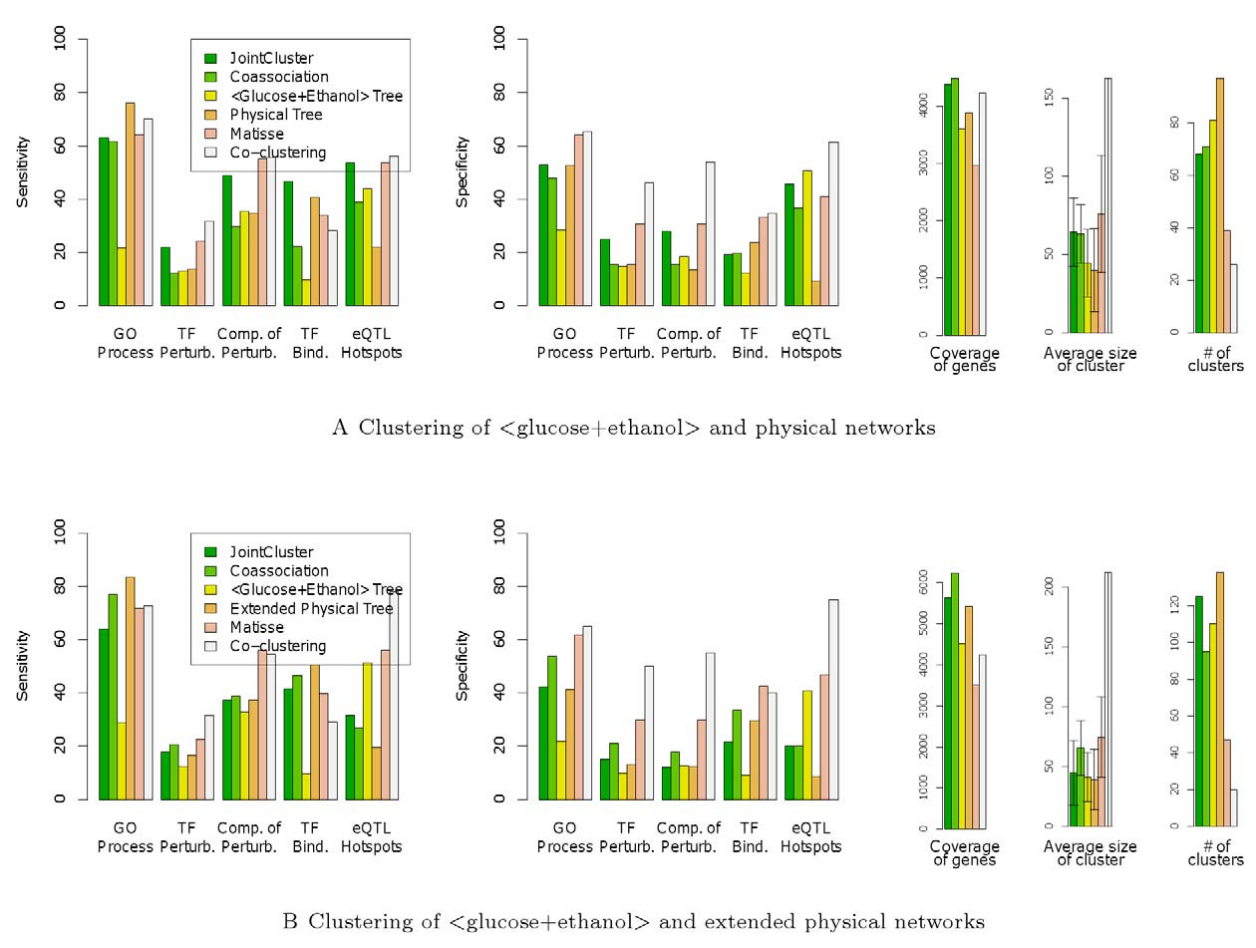
Comparison of JointCluster against Matisse, Co-clustering and other alternatives. The results on clustering the 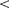
glucose+ethanol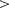 network with the (A) physical and (B) extended physical network are shown. The extended physical network contains several extra “back” genes (and associated interactions) that are not themselves expression profiled but increase physical connectivity among the profiled genes. The figure format is same as in Figure 3 , and coverage in (B) includes both back and profiled genes.

To compare JointCluster against methods that integrate only a single coexpression network with a physical network, such as Matisse and Co-clustering, we considered joint clustering of a combined 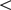
glucose+ethanol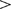 coexpression network and the physical network. The 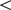
glucose+ethanol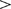 network refers to the single coexpression network built from expression data that is obtained by concatenating the normalized expression profiles of genes under the glucose and ethanol conditions. The results of different methods on this two-network clustering is in Figure 4 A. Since our results focus on clusters with at least 10 genes, we set the minimum cluster size parameter in Matisse to 10 (from its default 5). All other parameters of Matisse and other competing methods were set at the default values. The default size limit of 100 genes for Matisse clusters was used for JointCluster as well to enable a fair comparison. Co-clustering didn't have a parameter to directly limit cluster size. Despite setting its parameter for the number of clusters at 45 to get an expected cluster size of 100, Co-clustering detected very few (26) clusters of size at least 10 genes, half of which were large with more than 100 genes (including one coarse cluster with more than 800 genes). So Co-clustering achieves greater specificity than other methods (Figure 4 A) at the expense of a coarser clustering comprising few large clusters.

JointCluster has sensitivity and specificity that is comparable or slightly lower than Matisse across all reference classes except TF Binding Sites. However, JointCluster produces clusters that cover significantly more genes than Matisse (4382 vs 2964 genes respectively; see also Figure 4 A). Matisse assumes that the physical network is of better quality, and searches for coexpression clusters that are *each* connected in the physical network. This connectivity constraint excludes genes whose physical interactions are poorly studied or untested. JointCluster does not use such a constraint when parsing the clustering tree into clusters, and hence identifies clusters supported to varying extents in the two networks, including ones with weak support in the physical network. This could be a huge advantage in organisms such as human and mouse where the knowledge of physical interactions is far less complete than in yeast, especially for interactions that are tissue-specific or condition-specific.

The extreme examples among the roughly 1500 genes excluded by Matisse clusters were the 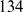 physically isolated genes (i.e., genes that do not interact with any of the other 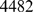 profiled genes in the physical network). JointCluster used connectivity in the coexpression network to include 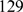 physically isolated genes in its clusters, and 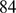 of these genes were significantly correlated (Pearson's correlation test 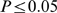, Bonferroni corrected for the cluster size) with more than half of the genes in their assigned cluster. Figure 4 in Supplementary Text S1 shows example coexpression clusters identified by JointCluster despite the poor physical connectivity among the isolated/other genes within the clusters.

The earlier study on Matisse extended physical connectivity within clusters by adding extra genes called “back” genes and their interactions to the physical network [9]. The physical interactions of back genes serve to better connect the (expression) profiled genes in the physical network, but the back genes' expression data is not used (or not available) for analysis. The results of integrating this extended yeast physical network, with 1774 extra back genes and 22,330 extra interactions, with the 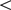
glucose+ethanol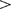 coexpression network is in Figure 4 B. The clusters of JointCluster covered a large fraction of genes, comprising 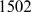 back and 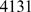 profiled genes, but they showed poor specificity due to the inclusion of several back genes with no expression information. Matisse on the other hand was specially designed to exploit a few of these back genes as needed to enhance physical connectivity, so it showed better sensitivity and specificity at a coverage of 182 back and 3327 profiled genes. Though back genes helped increase Matisse's coverage of profiled genes, Matisse clusters still missed several of the 4482 profiled genes. Considering the results before and after extension of the physical network, we see that the inclusive criteria used in JointCluster is preferable when the integrated physical network is not comprehensive.

### Contribution of individual and decomposed networks

#### Contribution of the physical network

**Figure 5:**
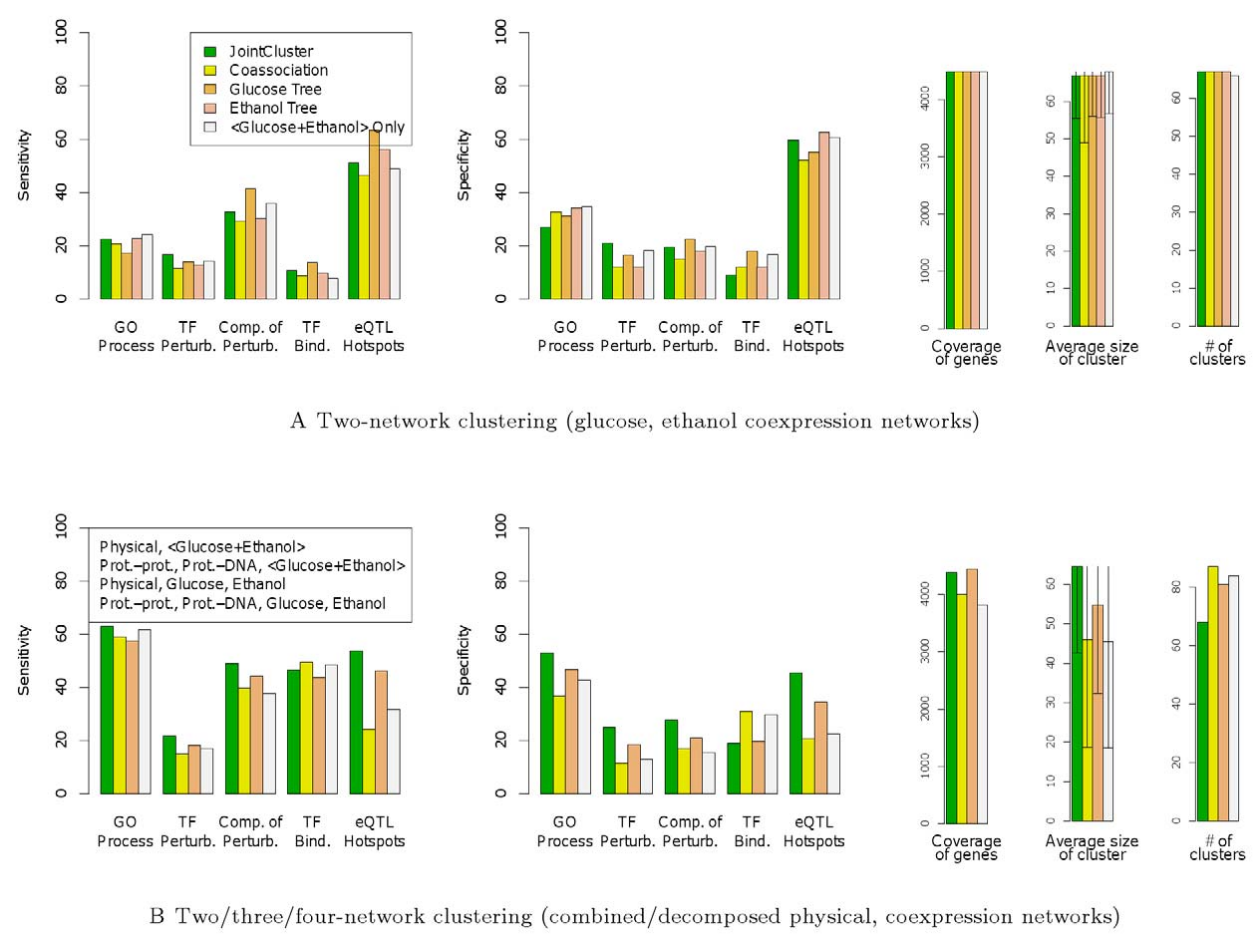
Contribution of individual and decomposed networks. (A) Contribution of the physical network can be assessed by comparing performance of JointCluster in this figure with that in Figure 3 B, and (B) Effect of decomposing the combined 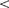
glucose+ethanol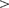 network into separate glucose and ethanol coexpression networks, or the physical network into protein-protein and protein-DNA networks, or both on the clusters detected by JointCluster on the physical and coexpression networks. The figure format is same as in Figure 3, and “prot.” refers to protein.

Addition of a physical network to the clustering of just the two coexpression networks (please compare Figures 3 B and 5 A) improved JointCluster's sensitivity, specificity or both for all reference classes but eQTL Hotspots. This improvement is most pronounced for GO Process and TF Binding Sites as expected. For the two independent perturbation classes, sensitivity improved with the addition of the physical network. These results are concordant with JointCluster's detection of subtle clusters that had better support in the physical network than the coexpression networks (eg. subtle clusters #28, #52, #54 discussed above). The different methods on jointly clustering the two coexpression networks showed similar performance, and also performed comparably to the single graph method, 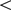
Glucose+Ethanol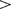 Only (Figure 5 A).

#### Performance on decomposing combined networks

The flexible framework of JointCluster in handling multiple networks allows easy experimentation with decomposing a combined network into its constituent networks before clustering them. For instance, in the joint clustering of 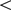
glucose+ethanol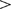 network and the physical network, the former can be decomposed into glucose and ethanol coexpression networks built from the corresponding expression data, and the physical network can be separated based on interaction type into protein-protein and protein-DNA networks. Figure 5 B shows results obtained using the decomposed constituent networks in place of a combined network, along with results for the combined networks. The clustering tree was produced and parsed as before, except for the use of a slightly adapted min-modularity score whenever protein-protein and protein-DNA networks were involved as described in Supplementary Methods in Text S1 .

Using glucose/ethanol networks in place of the single 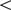
glucose+ethanol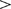 coexpression network when clustering them together with the physical network leads to comparable or slightly decreased performance for JointCluster (Figure 5 B, first and third bar). The expression data underlying the glucose/ethanol coexpression networks were obtained from the same lab using experiments repeated alongside on the same 109 segregants grown in as similar conditions as possible, except for the carbon source difference (glucose or ethanol) [14]. So it is not surprising to see comparable enrichment results before/after decomposing the combined coexpression network. The separation of the physical network into protein-protein and the protein-DNA networks leads to a much larger drop in specificity though (Figure 5 B, first two bars). This analysis revealed that JointCluster's three-network clusters supported by the physical, glucose and ethanol networks are similarly or more consistently enriched than alternative ways of decomposing the input networks.

## Discussion

Heterogeneous large-scale datasets capturing diverse aspects of the biology of a cell are accumulating at a rapid pace, and efforts to integrate them into a coherent view of cell regulation are intensifying. This integration could greatly facilitate a genome-wide model of the cell that could predict cellular response to various environmental and genetic perturbations (eg. [36]). The simultaneous clustering algorithm proposed here provides a versatile approach to integrating any number of heterogeneous datasets that could be represented as networks among genes, and summarizes the result as a collection of clusters supported by multiple networks.

Since its early applications to classifying cancer subtypes [37], clustering has rapidly become a standard analysis of expression datasets. We believe that simultaneous clustering is a natural progression in the application of clustering from single to multiple expression and interaction datasets. We demonstrated the utility of a combined analysis by applying our JointCluster algorithm on simulated and empirical datasets. We found the clusters produced by JointCluster on yeast physical and glucose/ethanol coexpression networks to be comparably or more consistently enriched for reference classes that reflect various aspects of yeast biology, in comparison to other methods of integrating the networks. Further, JointCluster can handle multiple heterogeneous networks, and hence more flexible than two-network clustering methods such as Matisse that search for coexpression clusters that are each connected in the physical network. This flexibility enables JointCluster to yield better coverage of genes, and to be broadly applicable in human or other organisms where the knowledge of physical interactions is less complete than in yeast. In the future, the framework could be extended to scale networks of different interaction types by different factors before integration.

Simultaneous clustering offers an unsupervised and exploratory approach to data integration, and hence complementary to supervised approaches that train machine-learning models on multiple data types to make directed predictions. Such supervised approaches could integrate different data types to predict functional linkages between gene pairs (see [38], [39] and references therein), or protein complexes using a training set of known complexes [40]. Though our method is not directed to predict complexes, the joint physical and coexpression clusters we found were enriched for reference protein complexes in MIPS. More importantly, the unsupervised fashion in which we parsed the joint clustering tree recovered functionally coherent pathways other than complexes. Simultaneous clustering is also complementary to unsupervised approaches that identify spectral patterns (not modules) shared between similarity graphs based on gene expression or TF binding data [41], or identify modules from paired datasets such as gene expression and drug responses profiled in the same cell lines [42].

The clusters detected by JointCluster from the yeast physical and expression datasets are consistent with known biology, and importantly extend our knowledge by highlighting biological processes, such as ribosome biogenesis, that may not have been completely characterized despite intense efforts to dissect them. The tangible value of a combined analysis is evident from the systematic evaluation of the clusters, and the case studies presented in this work. The intangible benefit of seeking support in the multiple networks considered in this study is the ready interpretation provided by the protein-protein and protein-DNA interactions within a cluster, in explaining the coordinate transcription of the cluster.

## Methods

The contribution of our work is an extension of a clustering framework for a single graph to jointly cluster multiple graphs. This section describes our simultaneous clustering framework in detail. Please refer Supplementary Methods in Text S1 for the algorithm analysis and more details on the overall JointCluster method and evaluation procedures.

### Single graph clustering review [15]

Consider a graph 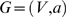, where 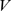 is the set of nodes and 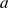 is a non-negative edge weight function. The weight 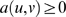 for any node pair 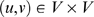 could for instance quantify the connection strength or similarity between the two nodes; note that a sparse graph would've many zero weight edges. For convenience, let us denote the total weight of any edge set 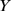 by 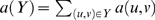. Similarly for any node sets 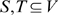, let 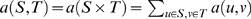, and 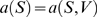. Using these notations, the total edge weight in the graph is 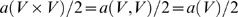. Also for singletons, 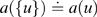 sums up the weights of edges incident at node 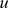.

The *conductance of a cut* 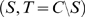 in a node set 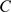, measured using the function 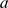, is defined as 
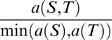
 (with the convention that this ratio is zero if its denominator is zero, since the numerator is also zero then). By normalizing the sum of edge weights 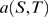 crossing the cut, the definition captures an intuitive notion of connectivity that is robust and invariant to scaling the edge weight function 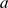 by any constant. To illustrate the intuitive notion, consider a cut separating a single node 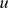 from other nodes in a cluster 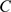. If the conductance of this cut is high, then a large fraction of all edges incident at node 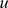 ends at another node in the cluster. Extending this notion, if the conductance of all cuts in 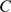 are high, the nodes in 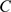 are robustly connected together. So the *conductance of a cluster* 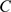 is defined as the minimum conductance of any cut in the cluster, and the *conductance of a clustering* or partition of 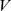 is the minimum conductance of any cluster in the partition.

When maximizing the conductance of the partition, it is desirable to control the cost of the inter-cluster edges as well. Let 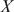 denote the inter-cluster edges, i.e., unordered node pairs 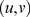 where 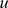 and 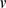 belong to different clusters in the partition. An 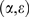 clustering of 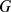 is a partition of its nodes into clusters such that

- the conductance of the clustering is at least 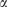, and
- the total weight of the inter-cluster edges 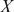 is at most an 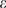 fraction of the total edge weight in the graph; i.e., 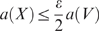.

We outline the approximate-cluster algorithm and its guarantees presented in [15]. The algorithm finds a cut approximating the *sparsest cut* (cut of minimum conductance) in the graph and recurses on both the pieces induced by this cut. Since finding the sparsest cut in a graph is a NP-hard problem, an approximation algorithm for the problem is used. Note that clustering a graph by minimizing 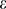 for a given 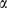 is also NP-hard by a reduction from the sparsest cut problem. The repeated removal of sparsest cuts is done until the pieces or clusters become well connected with no sparse cuts left in them. Making repeated cuts to partition a graph is strategically similar to a method on clustering an expression dataset [43], but that method works with minimum cuts rather than sparsest cuts. Sparsest cut is preferable in our context of heterogeneous datasets, since it minimizes the normalized measure of conductance.

To formalize the guarantees on the approximate-cluster algorithm, let 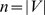 denote the number of nodes in the graph, and let the sparsest cut of conductance 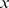 be approximated by a cut of conductance at most 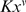 (where 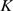 is independent of 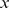, and 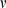 is a constant between 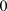 and 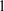). For instance, there are algorithms to find a cut of conductance at most 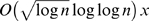 using metric embedding techniques [44] (all logarithms in this paper are to base two), or 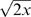 using efficient spectral techniques [15], [45] (our implementation uses spectral techniques).

#### Theorem 1

*If* 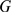 *has an* 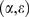 *clustering, then the approximate-cluster algorithm will find a clustering of quality* 
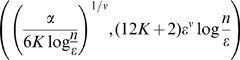
.

### Simultaneous clustering problem

Consider 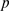 graphs 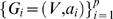 over the same nodes 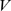 and different non-negative edge weight functions 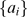. An 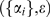 simultaneous clustering of the graphs is a partition of the nodes 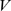 such that

- the conductance of the clustering is at least 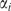 in graph 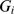 for all 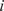, and
- the total weight of the inter-cluster edges 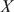 is at most an 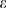 fraction of the total edge weight in all graphs; i.e., 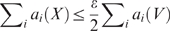.

The conductance thresholds 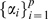 are graph-specific to enable search for clusters of varying quality in heterogeneous graphs. A natural approach to the inter-cluster edge cost is the sum of graph-specific costs 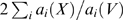, however it's a special case of the above aggregated cost 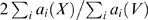 when each edge weight function 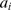 is scaled by a constant 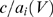. Note how these scalings, which our implementation employs, set the total edge weight of each graph to the same value 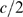 without changing the conductance value of cuts. This scale-invariance of conductance comes from the normalization factor in its definition as mentioned before.

### JointCluster algorithm and guarantees

The problem of minimizing the inter-cluster edge cost 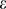 given a set of conductance thresholds 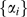 is NP-hard by reduction from the single graph case. We now present our basic algorithm, *JointCluster*, to simultaneously cluster multiple graphs, along with certain approximation guarantees on the quality of the clustering produced.

The algorithm starts with 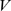 as the current node set. For each graph, the algorithm finds an approximate sparsest cut in the current node set, using the graph-specific edge weight function to measure conductance. The algorithm chooses among them any cut that is *sparse enough* as defined below, and recurses on the two pieces (node sets) induced by this cut. If no cuts get chosen for the current node set, the node set is output as a well connected cluster in all graphs. The cut approximating the sparsest cut in the current node set in 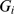 is *sparse enough* if the conductance of the cut, measured using the edge weight function 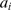, is at most 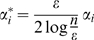.

To provide formal guarantees on the clustering produced by this algorithm, let 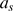, 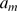 denote the respective edge weight functions of a sum and a min graph obtained from the multiple graphs. That is, for every edge 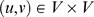, 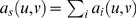 and 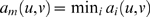. As before, let 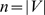 be the number of nodes, 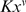 the approximation guarantee of the sparsest cut algorithm, and 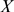 the inter-cluster edges of a given partition. We analysed our JointCluster algorithm (see Supplementary Methods in Text S1 ) to prove this theorem:

#### Theorem 2

*Let the graphs* 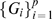 *admit an* 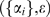 *simultaneous clustering, i.e., a partition of the common node set* 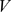 *that has conductance at least* 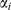 *in* 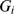 *and inter-cluster edge cost* 
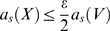
 *. Then, the JointCluster algorithm will find a partition of* 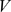 *that has conductance at least* 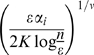 *in* 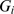 *and inter-cluster edge cost in the min graph* 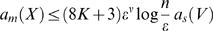.

### JointCluster heuristics and implementation

The theoretical guarantees of JointCluster algorithm are further augmented by effective heuristics and efficient implementation in practice.

#### Scaling heuristic description

The basic algorithm approximates the sparsest cut in each graph and chooses one of these cuts to recurse further. Since the chosen cut is sparse in the graph yielding the cut, we are able to bound the edges crossing the cut in this graph, but not necessarily the other graphs. In order to control the edges discarded in all graphs, we could approximate the sparsest cut in the sum graph, whose edge weight function 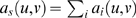. Working with the sum graph alone would yield coarse clusters though (eg. clusters well connected in some graphs but split into smaller clusters in the rest are not refined further). So we employ a heuristic that starts with the sum graph to control edges lost in all graphs, and transitions through a series of *mixture graphs* that approach the individual graphs to refine the clusters. A mixture graph 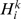 for a given scale 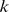 is a scaled sum graph with the edge weight function 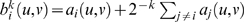 for every edge 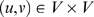. The scaling heuristic starts with 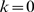 to work with the sum graph (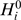 for any 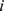 is the sum graph), and increments 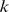 until it reaches a large value (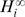 is the individual graph 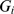).

**Figure 6:**
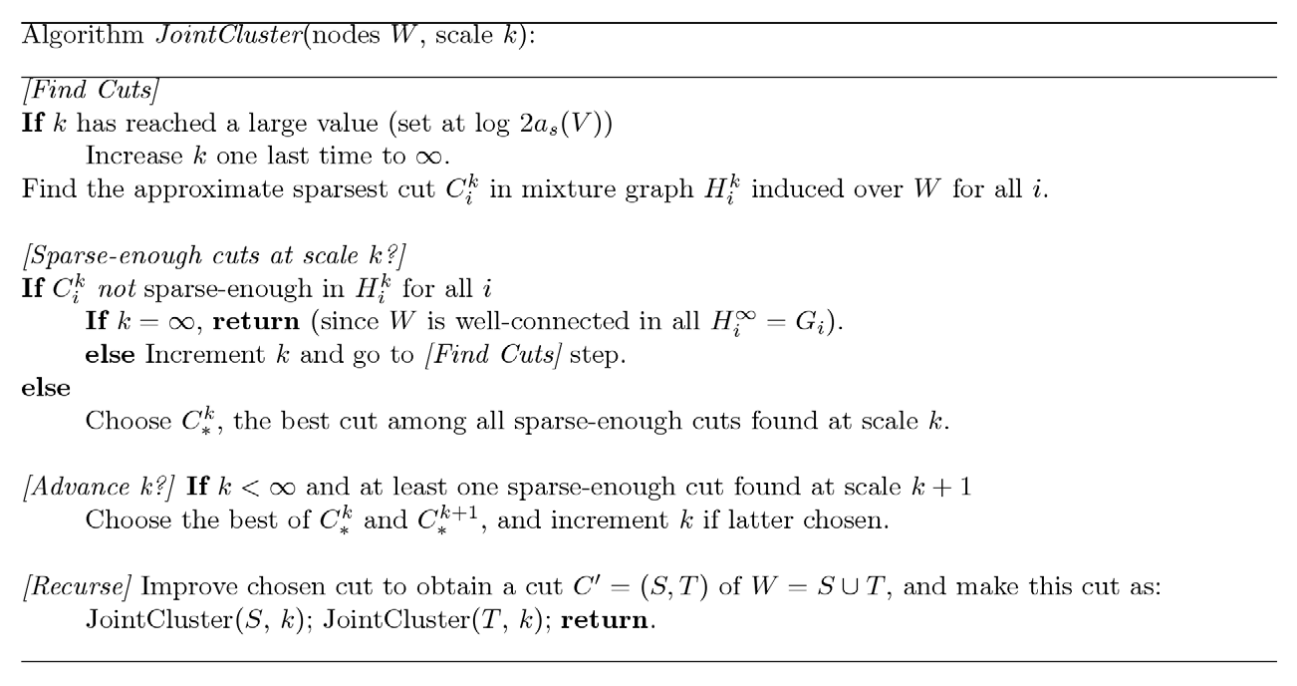
Pseudocode of JointCluster algorithm. The steps performed by JointCluster to find the clustering tree supported by multiple graphs is described here, using concepts defined in Methods section. The cuts made by the call *JointCluster* 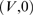 using graphs 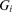 (defined over the same set of nodes 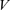 for all 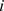) yield the simultaneous clustering tree.

The main step of the heuristic is finding the approximate sparsest cuts in mixture graphs 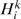 for all 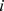 at the current scale 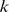, and choosing the *best* of these cuts that are *sparse-enough* to recurse further. A cut found in a mixture graph 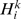 is *sparse-enough* (in 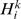) if the cut's conductance in 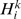 is less than 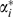. Similarly a cut is sparse-enough in an input graph 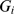 if the cut's conductance in 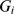 is less than 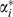. The *best* cut among many cuts is the one that is sparse-enough in the most number of input graphs, breaking ties toward the cut with the least fraction of cut edges in the input graphs (i.e., breaking ties toward the cut 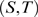 that minimizes 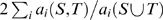). In these definitions, a input or mixture graph refers actually to a subgraph of the graph induced over the current nodeset 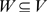 in the recursion call. So the approximate sparsest cut and conductances of cuts are found in induced subgraphs whose nodeset is 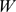 and edgeset includes only the edges with both endpoints in 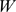 (this would imply for instance that 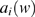 is 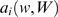 in the induced subgraph but 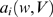 in the entire graph 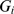). The pseudocode of our algorithm and heuristics is provided in Figure 6 . Improving the chosen cut in the pseudocode refers to improvement of its conductance using a flow-based algorithm [46].

#### Overall framework and running time in practice

The JointCluster algorithm and heuristics described above produces a clustering tree from a combined analysis of multiple graphs. This clustering tree is then parsed using a min-modularity score to produce clusters preserved in multiple graphs. The algorithm parameters such as the 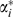 thresholds used in practice are also learnt automatically from the input graphs, so that we could jointly cluster multiple graphs in an unsupervised fashion. Complete information on the parsing of the clustering tree and learning of the parameters is provided in Supplementary Methods in Text S1.

The overall framework of JointCluster including the learning of parameters, producing the clustering tree and parsing it into clusters was time-efficient in practice. For instance, the running time for joint analyses of yeast networks defined over 4482 genes (two/three/four-network clusterings done in this study) all took about 30–70 minutes on a 2.53 GHz Linux machine. Please see Supplementary Text S1 for the availability of the software implementing the overall JointCluster framework.

## Supporting Information

Text S1 Supplementary Text S1 containing Supplementary Methods/Data/Figures/Tables.
(0.24 MB PDF)
