# Supplementary material for: Simultaneous Clustering of Multiple Gene Expression and Physical Interaction Datasets
Source: PLoS Comput Biol. 2010 Apr 15;6(4):e1000742. doi: 10.1371/journal.pcbi.1000742 (PMC2855327; doi:10.1371/journal.pcbi.1000742)
Supplement: Text S1 — Supplementary Text S1 containing Supplementary Methods/Data/Figures/Tables. (0.24 MB PDF) [file pcbi.1000742.s001.pdf]

Supplementary Text$^\diamond$ for "Simultaneous clustering of multiple gene expression and physical interaction datasets"

Manikandan Narayanan[1,a,*], Adrian Vetta[2], Eric E. Schadt[1,b], Jun Zhu[1,c,*]

1 Department of Genetics, Rosetta Inpharmatics (Merck), Seattle, WA, USA

2 Department of Mathematics and Statistics, and School of Computer Science, McGill University, Montreal, QC, Canada

* E-mail: manikandan_narayanan@merck.com (MN), junzhu_99@yahoo.com (JZ)

a Current address: Merck Research Labs, Boston, MA, USA

b Current address: Pacific Biosciences, Menlo Park, CA, USA

c Current address: Sage Bionetworks, Seattle, WA, USA

$^\diamond$ Referred to as (Supplementary) Text S1 in the main text.

1 Supplementary Methods

1.1 Algorithm Analysis

The proof of the theorem on JointCluster algorithm is organized in two parts: the first collects claims about the cuts made by the algorithm, and the second uses them to prove the theorem. Please refer the main text for the algorithm description, theorem statement, and relevant definitions.

Claims and notations. Let the cuts produced by the algorithm be (S_1, T_1), $(S_2, T_2), \ldots$, and let the *type* of a cut (S_j, T_j) be the label $\tau_j \in \{1, 2, \ldots, p\}$ of the graph that produced it. That is, (S_j, T_j) is the smallest of the chosen cuts in the algorithm description above, and approximates the sparsest cut in the current node set in G_{τ_j}. For convenience, let S_j be the "smaller" side of the cut (i.e., $a_{\tau_j}(S_j) \leq a_{\tau_j}(T_j)$). Slightly modify the algorithm just for the sake of analysis so that at any point, the current node set $S_j \cup T_j$ satisfying $a_{\tau_j}(S_j \cup T_j) < \frac{\epsilon}{n} a_{\tau_j}(V)$ is split into singletons. The conductance of singletons is defined to be 1.

Let a partition $C_1, C_2, \ldots, C_l$ of V be an $(\{\alpha_i\}, \epsilon)$ simultaneous clustering. We need to show that the partition defined by the algorithm's cuts approximates this optimal clustering. To this end, note that the inter-cluster edges X of the algorithm's partition is simply the edges crossing all cuts $\{(S_j, T_j)\}$. To bound the cost of edges lost in some (S_j, T_j), we define a new *cluster-avoiding* cut (S'_j, T'_j) in $S_j \cup T_j$ as follows. For each cluster C_t in the optimal clustering, place all of $(S_j \cup T_j) \cap C_t$ either into S'_j if $a_{\tau_j}(S_j \cap C_t) \geq a_{\tau_j}(T_j \cap C_t)$, or into T'_j otherwise. By this construction, $|a_{\tau_j}(S_j) - a_{\tau_j}(S'_j)| \leq \sum_{t=1}^{l} \min(a_{\tau_j}(S_j \cap C_t), a_{\tau_j}(T_j \cap C_t))$.

We now present two claims that will help us bound the edges lost in the algorithm's cuts in the next section. These claims are simple extensions of the single graph case [1], but proved here for completeness. The first claim is on the maximum number of S_j or S'_j sets a node can belong to.

Claim 1. *Consider any graph G_i. For each node $u \in V$, there are at most $\log \frac{n}{\epsilon}$ values of j such that $\tau_j = i$ and u belongs to S_j. Further, there are at most $2 \log \frac{n}{\epsilon}$ values of j such that $\tau_j = i$ and u belongs to S'_j.*

Proof of Claim 1. Fix a node $u \in V$. Let

$$I_u = \{j : u \in S_j,\, \tau_j = i\}, \qquad J_u = \{j : u \in S'_j \setminus S_j,\, \tau_j = i\}\ .$$

Clearly if $u \in S_j \cap S_k$ with $j < k$, then (S_k, T_k) must be a partition of S_j or a subset of S_j. Also, if the cut types $\tau_j = \tau_k = i$, we have $a_i(S_k) \leq \frac{1}{2} a_i(S_k \cup T_k) \leq \frac{1}{2} a_i(S_j)$. So $a_i(S_j)$ reduces by a factor of 2 or greater between two successive times $j \in I_u$. The maximum value of $a_i(S_j)$ is at most $a_i(V)$ and the minimum value is at least $\frac{\epsilon}{n} a_i(V)$, yielding the first statement of the claim.

Now suppose $j, k \in J_u$ with $j < k$. Suppose also $u \in C_t$. Then $u \in T_j \cap C_t$, and T_j or a subset of T_j is later partitioned into (S_k, T_k). Since $u \in S'_k \setminus S_k$ with $\tau_k = i$, we also have $a_i(T_k \cap C_t) \leq a_i(S_k \cap C_t)$. Thus $a_i(T_k \cap C_t) \leq \frac{1}{2} a_i((S_k \cup T_k) \cap C_t) \leq \frac{1}{2} a_i(T_j \cap C_t)$. This halving of $a_i(T_j \cap C_t)$ between two successive times that $j \in J_u$ shows that $|J_u| \leq \log \frac{n}{\epsilon}$. This proves the second statement in the claim as $u \in S'_j$ when $u \in S_j$ or $u \in S'_j \setminus S_j$. □

The second claim considers a set C_t in the optimal partition, and uses its high conductance (of at least α_i in G_i) to show that another conductance-like measure defined using intra-cluster cut edges (within C_t) is high as well.

Claim 2. *Consider any graph G_i. For each cluster C_t in the optimal clustering, which has conductance at least α_i in G_i, we have*

$$\sum_j a_i(S_j \cap C_t, T_j \cap C_t) \geq \frac{\alpha_i}{\log \frac{n}{\epsilon}} \sum_j \min(a_i(S_j \cap C_t), a_i(T_j \cap C_t)) \ .$$

Proof of Claim 2. Consider how the cuts (S_1, T_1), $(S_2, T_2), \ldots$ induce a partition of C_t into $P_1, P_2, \ldots$. Every edge between two vertices in C_t that belong to different sets of the partition must be cut by some cut (S_j, T_j), and conversely, every edge of every cut $(S_j \cap C_t, T_j \cap C_t)$ must have its two end points in different sets of the partition. So given that C_t has conductance α_i, we obtain

$$\begin{aligned} \sum_{\text{all } j} a_i(S_j \cap C_t, T_j \cap C_t) &= \frac{1}{2} \sum_{\text{all } s} a_i(P_s,\, C_t \setminus P_s) \\ &\geq \alpha_i \sum_s \min(a_i(P_s),\, a_i(C_t \setminus P_s)) \ . \end{aligned}$$

Each node $u \in C_t$ belongs to the smaller (with respect to $a_i(\cdot)$) of the two sets $S_j \cap C_t$, $T_j \cap C_t$ for at most $\log \frac{n}{\epsilon}$ values of j. Also every P_s contained in (and which partition) the smaller of $S_j \cap C_t$, $T_j \cap C_t$ is smaller than $C_t \setminus P_s$. So we have

$$\sum_j \min(a_i(S_j \cap C_t), a_i(T_j \cap C_t)) \leq \log \frac{n}{\epsilon} \sum_s \min(a_i(P_s),\, a_i(C_t \setminus P_s)) \ .$$

The claim follows from these two bounds taken together. □

Proof of algorithm guarantees. We now prove that the partition defined by the algorithm's cuts approximates the optimal clustering, which has conductance at least α_i in G_i and inter-cluster edge cost at most ϵ in the sum graph (V, a_s).

Proof of Theorem 2 in the main text. From the algorithm description, it follows that each cluster output by our algorithm upon termination has conductance at least

$$\left(\frac{\alpha_i^*}{K}\right)^{1/\nu} = \left(\frac{\epsilon\, \alpha_i}{2K \log \frac{n}{\epsilon}}\right)^{1/\nu}$$

in G_i for all i.

Thus it remains to bound the weight of the inter-cluster edges. To do so, we divide the algorithm's cuts into two groups, and bound their edge weights separately. The first group H consists of cuts with "high" conductance within clusters. Let $a_i^{\mathrm{I}}(S_j, T_j)$ denote the weight of intra-cluster edges of the cut (S_j, T_j); i.e., $a_i^{\mathrm{I}}(S_j, T_j) = \sum_{t=1}^{l} a_i(S_j \cap C_t, T_j \cap C_t)$. Then,

$$H = \Big\{ j : a_{\tau_j}^{\mathrm{I}}(S_j, T_j) \geq 2\alpha_{\tau_j}^* \sum_{t=1}^{l} \min(a_{\tau_j}(S_j \cap C_t), a_{\tau_j}(T_j \cap C_t)) \Big\}.$$

The remaining cuts will be called as cuts with "low" conductance within clusters.

We now bound the cost of the high conductance group using the cost of the cluster-avoiding cuts. Recall that a cluster-avoiding cut (S'_j, T'_j) of a cut (S_j, T_j) satisfies $|a_{\tau_j}(S_j) - a_{\tau_j}(S'_j)| \leq \sum_t \min(a_{\tau_j}(S_j \cap C_t), a_{\tau_j}(T_j \cap C_t))$. Also for all $j \in H$, we have

$$\begin{aligned}\alpha^*_{\tau_j} a_{\tau_j}(S_j) &\geq a_{\tau_j}(S_j, T_j)\\ &\geq a^{\mathrm{I}}_{\tau_j}(S_j, T_j)\\ &\geq 2\alpha^*_{\tau_j} \sum_t \min(a_{\tau_j}(S_j \cap C_t), a_{\tau_j}(T_j \cap C_t)).\end{aligned}$$

Putting them together, we obtain $|a_{\tau_j}(S_j) - a_{\tau_j}(S'_j)| \leq \frac{1}{2} a_{\tau_j}(S_j)$, and hence $\min(a_{\tau_j}(S'_j), a_{\tau_j}(T'_j)) \geq \frac{1}{2} a_{\tau_j}(S_j)$. We now use the sparsest cut approximation guarantee to upper bound $a_{\tau_j}(S_j, T_j)$ in terms of $a_{\tau_j}(S'_j, T'_j)$.

$$\begin{aligned}\frac{a_{\tau_j}(S_j, T_j)}{a_{\tau_j}(S_j)} &\leq K\Big(\frac{a_{\tau_j}(S'_j, T'_j)}{\min(a_{\tau_j}(S'_j), a_{\tau_j}(T'_j))}\Big)^{\nu}\\ &\leq K\Big(\frac{2\, a_{\tau_j}(S'_j, T'_j)}{a_{\tau_j}(S_j)}\Big)^{\nu}\end{aligned}$$

Hence we have bounded the overall cost of the high conductance cuts with respect to the cost of the cluster-avoiding cuts. Observe that every edge in a cluster-avoiding cut is an inter-cluster edge in the optimal clustering. Also note that if an edge (u, v) crosses a cut (S'_j, T'_j), then either $u \in S'_j$ or $v \in S'_j$. So by applying Claim 1 for any $i = 1, 2, \ldots, p$, each edge (u, v) crosses a cut (S'_j, T'_j) of type $\tau_j = i$ for at most $4 \log \frac{n}{\epsilon}$ values of j. Applying these observations along with Claim 1 and Holder's inequality, we bound the cost of the high conductance group cuts in the min graph (V, a_m).

$$\begin{aligned}\sum_{j \in H} a_m(S_j, T_j) &\leq \sum_{j \in H} a_{\tau_j}(S_j, T_j)\\ &\leq \sum_{j \in H} K \left(2a_{\tau_j}(S'_j, T'_j)\right)^{\nu} a_{\tau_j}(S_j)^{1-\nu}\\ &\leq K \left(\sum_{j \in H} 2a_{\tau_j}(S'_j, T'_j)\right)^{\nu} \left(\sum_{j \in H} a_{\tau_j}(S_j)\right)^{1-\nu}\\ &\leq K \left(\sum_{i=1}^{p} \sum_{j:\tau_j = i} 2a_i(S'_j, T'_j)\right)^{\nu} \left(\sum_{i=1}^{p} \sum_{j:\tau_j = i} a_i(S_j)\right)^{1-\nu}\\ &\leq K \left(4\epsilon \log \frac{n}{\epsilon} \sum_{i=1}^{p} a_i(V)\right)^{\nu} \left(\log \frac{n}{\epsilon} \sum_{i=1}^{p} a_i(V)\right)^{1-\nu}\\ &\leq 4K\epsilon^{\nu} \log \frac{n}{\epsilon} a_s(V)\end{aligned}$$

Next we deal with the group of cuts with low conductance within clusters. Using the definition of H

and Claim 2, we can bound the intra-cluster cost of the low conductance group of cuts.

$$
\begin{aligned}
\sum_{j \notin H} a^{\mathrm{I}}_{m}(S_j, T_j) &\leq \sum_{j \notin H} a^{\mathrm{I}}_{\tau_j}(S_j, T_j) \\
&\leq \sum_{j \notin H} 2\alpha^*_{\tau_j} \sum_{t=1}^{l} \min(a_{\tau_j}(S_j \cap C_t), a_{\tau_j}(T_j \cap C_t)) \\
&\leq \sum_{i=1}^{p} 2\alpha^*_i \sum_{t=1}^{l} \sum_{\text{all } j} \min(a_i(S_j \cap C_t), a_i(T_j \cap C_t)) \\
&\leq \sum_{i=1}^{p} 2\alpha^*_i \sum_{t} \frac{\log \frac{n}{\epsilon}}{\alpha_i} \sum_{j} a_i(S_j \cap C_t, T_j \cap C_t) \\
&\leq \epsilon \sum_{i=1}^{p} \sum_{j} a^{\mathrm{I}}_i(S_j, T_j) \\
&\leq \epsilon \sum_{i=1}^{p} \frac{a_i(V)}{2} \\
&= \epsilon \frac{a_s(V)}{2}
\end{aligned}
$$

In addition, since each inter-cluster edge in the optimal clustering belongs to at most one cut (S_j, T_j), the cost of such edges crossing the low-conductance group of cuts in the sum graph (and hence min graph) is at most $\frac{\epsilon}{2} a_s(V)$. So the total cost of the low conductance group of cuts in the min graph is $\epsilon\, a_s(V)$.

To get the total edge cost in the min graph, we then sum up the cost of the two groups of cuts from above, and note that splitting up all the $S_j \cup T_j$ with $a_{\tau_j}(S_j \cup T_j) \leq \frac{\epsilon}{n} a_{\tau_j}(V)$ into singletons costs us at most $\frac{\epsilon}{2}\, a_s(V)$ on the whole. Substituting $a_s(V)$ as twice the total sum of edge weights gives the edge cost bound in Theorem 2 in the main text. □

1.2 JointCluster – Overall Framework

JointCluster performs a combined analysis of multiple graphs using a flexible framework comprising two phases. The first phase produces a clustering tree using the JointCluster algorithm and heuristics described in the main text, and the second phase parses this clustering tree to produce clusters preserved in multiple graphs as described in this section. The algorithm parameters are also learnt automatically as described here, so that we could jointly cluster multiple graphs in an unsupervised fashion.

Parsing the clustering tree. Our algorithm recursively partitions the common node set V of the graphs. To capture this hierarchical structure, we use a clustering tree with the same structure as the algorithm's recursion call tree, and *map* the subset of V on which a recursive call is made to the corresponding vertex in the tree. Adopting an empirically tested clustering framework [2], our JointCluster method parses this clustering tree into a partition of V that both *respects* the clustering tree and optimizes a certain objective function.

A partition of V is a collection of disjoint node sets $C_1, C_2, \ldots C_T$ ($C_t \subseteq V$) whose union equals V. A collection of disjoint node sets is said to *respect* a clustering tree if each node set is mappable to some vertex in the clustering tree. Our objective function for a partition is based on the modularity score [3], studied in other biological contexts [4], and described next.

We take the *min–modularity* score of a partition C, which is the sum of the *min–modularity* score of the sets C_t in the partition, as our objective function. We compute the modularity score of C_t in each

graph G_i as below, and take their minimum as the *min–modularity* score of C_t. The modularity of a set of nodes C_t in graph G_i is given by: $Modularity_i(C_t) = \Big(a_i(C_t, C_t)/a_i(V) - (a_i(C_t, V)/a_i(V))^2\Big)$. The first term is the normalized edge density of the nodes in G_i and the second the expectation of the same measure in a randomized graph obtained from degree-preserved shuffling of the edges in G_i.

The partition of V that respects the clustering tree and optimizes the *min–modularity* score can be found by dynamic programming, much in the same way as done for the correlation clustering objective function in [2]. For a given vertex in the clustering tree, let $D \subseteq V$ be the nodes mapped to it, and D_l, D_r be the nodes mapped to its left and right children respectively. If $OPT(D)$ is the optimal partition of D, then the dynamic programming recurrence is $OPT(D) = \arg\max(\textit{min–modularity}(D), \textit{min–modularity}(OPT(D_l) \cup OPT(D_r)))$. To enable a fair comparison of JointCluster with other methods, we needed a size limit on the clusters. To find a partition where each set or cluster in the partition is of some maximum size (set at 100 nodes), we simply change the above recurrence to $OPT(D) = OPT(D_l) \cup OPT(D_r)$ whenever $|D| > 100$. From the size-limited optimal partition of V found using this procedure, we only output clusters of some minimum size (at least 10 nodes) as the final list of meaningful clusters. This list is ordered by the min-modularity scores of clusters (highest score first), and the clusters are identified by their rank in this ordering.

Parameter learning. In the problem definition, we saw how graph-specific conductance thresholds $\{\alpha_i\}$ accommodate the varying extents to which different graphs can be clustered. We desire an unsupervised method for learning the related conductance threshold α_i^* (see algorithm description) for each network of interest G_i from the inherent cluster structure present in the network. To do so, we repeat the following procedure separately for each graph G_i.

1. Separately cluster G_i using JointCluster with the conductance threshold set without loss of generality to the maximum possible value 1. The result is a fully-resolved clustering tree, where each node in G_i is mapped to a leaf vertex in the tree.

2. Parse the clustering tree into a clustering with the optimal min-modularity score, which turns out to be the modularity score as only one graph is clustered. This clustering yields a set of size-constrained, meaningful clusters $\{C_t\}$ as described above.

3. Set α_i^* to the minimum conductance threshold that would result in the same set of clusters $\{C_t\}$ upon clustering G_i as the threshold of 1. This value is determined from the clustering tree by selecting the tree vertices to which the clusters $\{C_t\}$ are mapped to, and taking the maximum of the conductance of all cuts made by the algorithm in resolving the clustering tree to the selected vertices.

Note that when clustering multiple graphs, very stringent conductance thresholds might not yield any (non-trivial) clusters, and very low conductance thresholds might only yield coarsely resolved clustering trees and large clusters. The rationale behind the above heuristic is to automatically choose a threshold that is both sufficiently low to enable simultaneous clustering with the scaling heuristic, and sufficiently high to provide a clustering tree that is at least as resolved as the single-graph clustering trees.

Significance of modularity scores. The modularity score of a cluster in a network is said to be statistically significant (or simply significant) if the P-value of the score is at most 1/100 as estimated from random sets containing the same number of genes as the cluster. In detail, a random set of genes of the same size as the cluster is chosen without replacement from all 4482 genes in the network, and its modularity score calculated in the network. The P-value of a modularity score a is calculated by the proportion of 100 random sets that achieve modularity score at least a. The P-value of the min-modularity score of a cluster is similarly estimated from the min-modularity score of 100 random sets of

genes of the same size as the cluster, and the min-modularity score is said to be (statistically) significant if the estimated $P < 1/100$. The P-value of the min-modularity score of a whole clustering is estimated from the min-modularity score of 100 random partitions of all genes into random sets of genes, with each random partition having the same number of clusters and cluster sizes as the whole clustering. Again, the min-modularity score of a clustering is said to be (statistically) significant if the estimated $P < 1/100$.

Adapted scoring for protein-protein and protein-DNA networks. To obtain some of our results, we decomposed the physical network into a network of protein-protein and a network of protein-DNA interactions, and applied JointCluster to these two networks in place of the single physical network. The clustering tree was produced and parsed as before, except for the use of a slightly adapted min-modularity score whenever protein-protein and protein-DNA networks were involved. The adapted min-modularity score of a cluster is the minimum of the best modularity score of the cluster in the protein-protein and protein-DNA networks, and the modularity score of the cluster in other integrated networks if any. This allowed us to find clusters that were present in the protein-protein network (as a protein complex for instance) or in the protein-DNA network (as a coregulated module for instance).

1.3 Performance Comparison

Tested methods. We already described the tested methods (JointCluster, single tree, single graph and Coassociation methods) in the main text. To complete the description, we provide details on implementing the spectral clustering method M [2], and building the coassociation graph [5]. The spectral method M was implemented by simply applying our JointCluster program on one input graph, since JointCluster is an extension of the spectral method from one graph to multiple graphs. Clustering a graph using this implementation of method M yields the clustering tree, and optionally the clusters that result from parsing this tree using the modularity score described above. The coassociation graph is basically a consensus of different clusterings (partitions) of the same set of nodes. The graph was defined over the nodes being clustered, with an edge between two nodes u, v weighted by the number of clusterings in which both u, v belong to the same cluster.

The implementation of Matisse and Co-clustering methods described in the main text was available as a single software `http://acgt.cs.tau.ac.il/matisse/`. The same software was also used to estimate missing expression values using the k-nearest neighbors option (with $k = 10$) before building the yeast coexpression networks.

Simulated data for benchmarking. To generate one random instance of two test graphs G_1, G_2, we proceeded as in an earlier study [4], by creating each graph over 128 nodes and implanting a fixed "true" clustering of 4 clusters with 32 nodes each. Let the internal degree of a node v count the neighbors of v that belong to the same cluster as v, and its external degree count the remaining neighbors of v. In order to obtain a meaningful cluster structure, the edge probability between two nodes was set such that the average external degree (the k_{out} parameter) of every node is at most its average internal degree. The sum of these two average degrees was fixed at a constant 16 as in the earlier study, so only the values $k_{out} \leq 8$ lead to a meaningful cluster structure. In more detail, we classified edges as intra or inter cluster, and set these edges independently with probability p_i and p_o respectively. The p_i and p_o values were chosen such that the average internal degree $32p_i$ and average external degree $k_{out} = 96p_o$ summed up to 16 for different values of k_{out}.

Reference classes. The biological significance of detected clusters were assessed using reference sets of yeast genes belonging to different classes, as mentioned in the Results. We provide detail on how these five reference classes were collected.

- *GO Process:* Genes in each reference set in this class are annotated either directly or indirectly to the same GO Biological Process term, using any evidence code other than IEA (Inferred from Electronic Annotation) [6] (Jan 2009 version). We restrict ourselves to sets of size at least 50 and at most 1000 to avoid reference sets that are either too small or too large to provide a meaningful enrichment category.
- *TF (Transcription Factor) Perturbations:* Genes in each set are differentially expressed at P-value at most 0.01 between TF-perturbed and wildtype (or control) strains. The list of reference sets from two studies, TF deletion [7] and TF over-expression [8], are concatenated to form this reference class. The P-value for differential expression of a gene under a TF deletion or overexpression is based respectively on the X-score or Z-score reported in the study.
- *Compendium of Perturbations:* Genes in each set are differentially expressed at P-value at most 0.05 and exhibit a fold change at least 1.5 between perturbed (deletions of genes, or chemical treatment) and wildtype (or mock-treated control) yeast strains [9]. The P-values based on a gene-specific error model and fold changes are as reported in the study.
- *TF Binding Sites:* Genes in a set have binding sites of the same TF in their upstream genomic regions, with sites predictions downloaded from a previous study based on ChIP binding data [10] at a binding P-value cutoff of 0.005 and cross-species conservation of site sequences determined using a moderate criterion [11].
- *eQTL Hotspots:* The genomic regions with wide-spread transcriptional effects, i.e., exhibiting a significant excess of linkages of expression-related traits to genotypic variations, are called eQTL hotspot regions. The hotspot regions have been reported for three traits, gene expression under glucose and ethanol conditions and difference between these two expressions, using linkages significant at about 5% FDR [12]. Genes with trait linkages to such a eQTL hotspot region are grouped into a reference set.

Besides the criteria described above, we discard from each class, reference sets that are too small (less than 5 genes) to provide a meaningful enrichment category. A reference set comprising differentially expressed genes is referred to as a signature set or simply signature in the text (eg. deletion, overexpression or perturbation signatures).

Evaluation measures. To evaluate the methods on clustering of the simulated datasets, we used the standard Jaccard index measure, which ranges from 0 to 1, to reflect the degree of overlap between the true clustering and the clustering detected by the methods. *Jaccard Index* between two clusterings of the same set of elements is the ratio between the number of element pairs that are intra-cluster wrt (with respect to) both clusterings and the number of element pairs that are intra-cluster wrt either of the clusterings. Given a clustering (partition) of a set of elements into clusters, a pair of elements that belong to a single cluster is denoted as *intra-cluster* wrt this clustering.

To evaluate the methods on clustering of the yeast networks, we measured the overlap between clusters detected by the methods against sets in the various reference classes. The significance of overlap between two sets of genes belonging to a larger background set (typically all the genes in the yeast networks) is reported as an *enrichment P-value* computed using the hypergeometric distribution (denoted as P in the text).

Sensitivity and specificity measure how well the clusters produced by a clustering method align with reference sets of a specific class such as GO Process or TF Perturbations. *Sensitivity* is the fraction of reference sets in the class that are enriched for genes belonging to some cluster output by the method. *Specificity* is the fraction of clusters output by a method that are enriched for genes belonging to some reference set in the class. Both fractions are reported in the tables as percentages rounded to the nearest tenth. A cluster is said to be *significantly enriched* or simply *enriched* for genes belonging to some reference set, if the enrichment P-value of overlap between the cluster and reference set genes, computed

as said above using all genes in the networks of interest as background, is at most 0.005 after Bonferroni correction for the multiple reference sets being tested. The same method is used to declare a reference set as being *enriched* for genes belonging to some cluster, but the Bonferroni correction is now using the number of clusters being tested.

A set of genes is said to be *recovered* by a clustering method if it is enriched for genes belonging to some cluster in the clustering found by the method, using the same definition of enrichment of a cluster for a reference set described above.

2 Supplementary Data

2.1 JointCluster: Results on Yeast Datasets and Software

The genes in the three-network clusters obtained from applying JointCluster on the yeast physical and glucose/ethanol coexpression networks is provided in an accompanying "`pge_clusters.orf`" file. The enrichment of these clusters for all five reference classes is provided in an accompanying "`pge_clusters_enrich.out`" file. The prediction of function for uncharacterized ORFs (Open Reading Frames) based on the GO Process enrichment of these clusters is provided in an accompanying "`pge_clusters_predns.out`" file. The accompanying files and JointCluster software are available at `http://www.math.mcgill.ca/vetta/jc/` .

References

1. Kannan R, Vempala S, Vetta A (2000) On clusterings - good, bad and spectral. In: Proc Annu IEEE Symp Foundations of Comput Sci (FOCS). pp. 367-377.
2. Cheng D, Vempala S, Kannan R, Wang G (2005) A divide-and-merge methodology for clustering. In: Proc ACM Symp Princ of Database Syst (PODS). pp. 196-205.
3. Brandes U, Delling D, Gaertler M, Gorke R, Hoefer M, et al. (2008) On modularity clustering. IEEE Trans Knowl Data Eng 20: 172–188.
4. Guimera R, Nunes Amaral LA (2005) Functional cartography of complex metabolic networks. Nature 433: 895-900.
5. Topchy AP, Jain AK, Punch WF (2003) Combining multiple weak clusterings. In: Proc IEEE Int Conf on Data Mining (ICDM). pp. 331-338.
6. Ashburner M, Ball CA, Blake JA, Botstein D, Butler H, et al. (2000) Gene Ontology: tool for the unification of biology. Nat Genet 25: 25-29.
7. Hu Z, Killion PJ, Iyer VR (2007) Genetic reconstruction of a functional transcriptional regulatory network. Nat Genet 39: 683-687.
8. Chua G, Morris QD, Sopko R, Robinson MD, Ryan O, et al. (2006) Identifying transcription factor functions and targets by phenotypic activation. Proc Natl Acad Sci U S A 103: 12045-12050.
9. Hughes TR, Marton MJ, Jones AR, Roberts CJ, Stoughton R, et al. (2000) Functional discovery via a compendium of expression profiles. Cell 102: 109-126.
10. Harbison CT, Gordon DB, Lee TI, Rinaldi NJ, Macisaac KD, et al. (2004) Transcriptional regulatory code of a eukaryotic genome. Nature 431: 99-104.
11. MacIsaac K, Wang T, Gordon DB, Gifford D, Stormo G, et al. (2006) An improved map of conserved regulatory sites for Saccharomyces cerevisiae. BMC Bioinformatics 7: 113.

12. Smith EN, Kruglyak L (2008) Gene-environment interaction in yeast gene expression. PLoS Biol 6: e83.

Supplementary Figures and Tables

This section contains the supplementary figures and tables referred to in the main text.

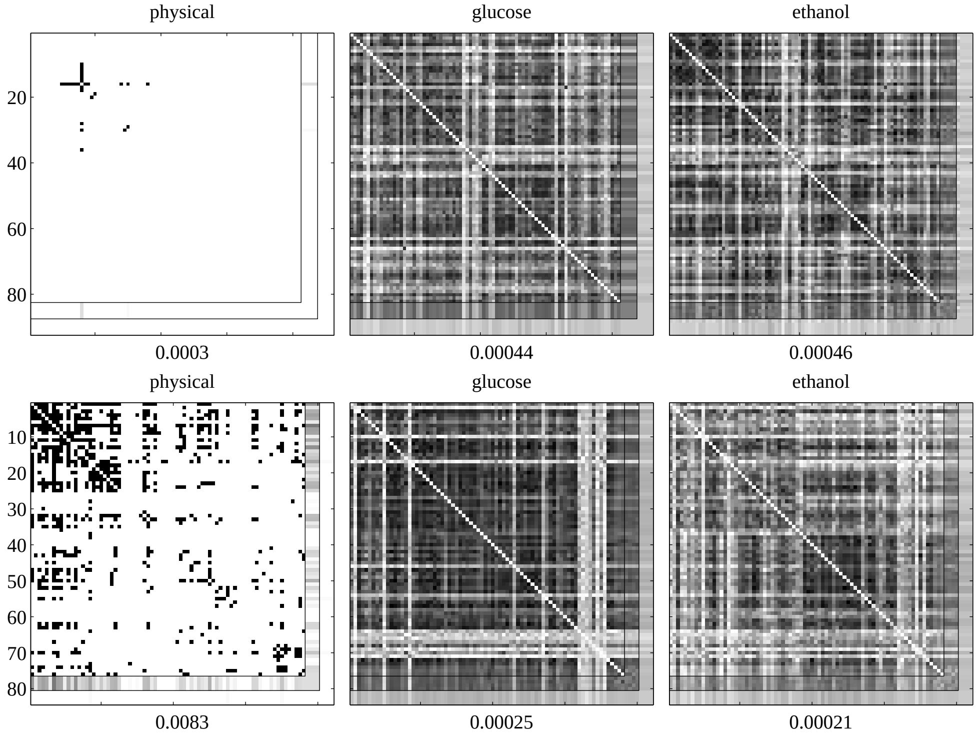

Figure 1. Connectivity of the best preserved clusters. Two top-ranking preserved clusters (Clusters #1, #2) detected from applying JointCluster on the yeast physical and glucose/ethanol coexpression networks. Cluster #1 is significantly enriched for functions related to translation and mitochondria, and Cluster #2 is enriched for functions related to ribosome biogenesis. Modularity score of a cluster in each network is shown below the corresponding heatmap. In these heatmaps, the darkness of a spot x, y is scaled by the weight of the edge between the x–th and y–th gene in the cluster (darkest spot is weight 1.0 and brightest is weight 0.0). The weight is a binary (1 or 0) indicator of interaction between the genes in the physical network (labeled physical), and absolute correlation coefficient between genes in the coexpression network (labeled glucose and ethanol). The inner band enveloping a heatmap shows for each node v, the average edge weight from v to neighbors of v belonging to the same cluster as v; the outer band shows the average edge weight from v to the remaining neighbors of v. Hence, a good cluster's inner band would be darker than its external band. The weight is a binary (1 or 0) indicator of interaction between the genes in the physical network (labeled physical), and absolute correlation coefficient between genes in the coexpression network (labeled glucose/ethanol).

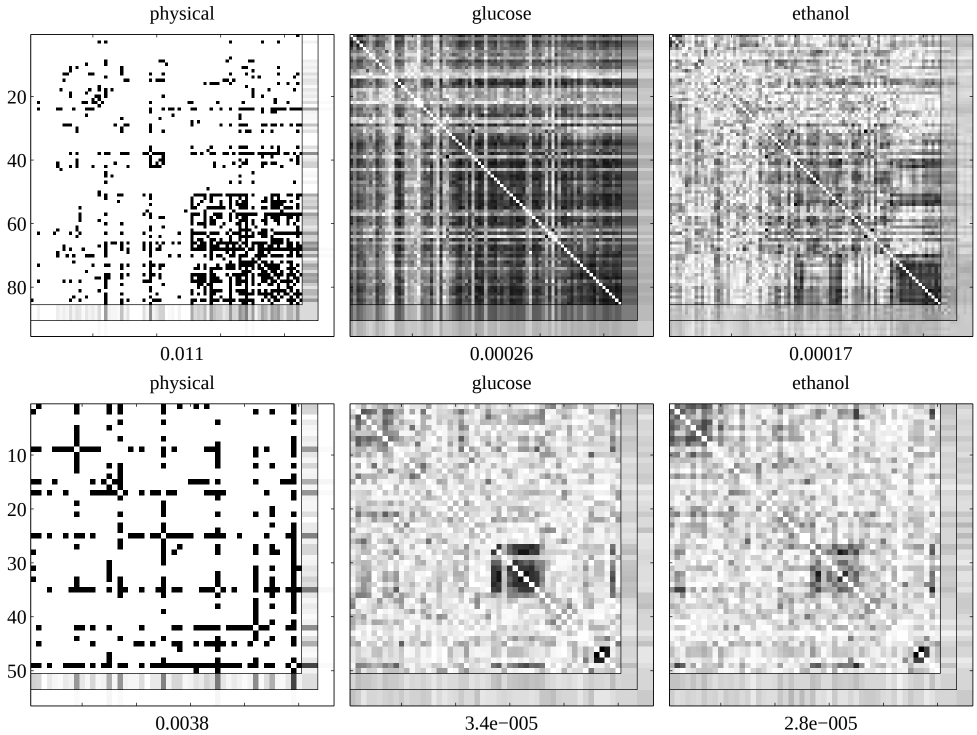

Figure 2. Among the biologically significant clusters enriched for all reference classes, the clusters with the highest (Cluster #4 in top row) and the lowest (Cluster #28 in bottom row) min-modularity score are shown here. Heatmap conventions are the same as in Figure 1.

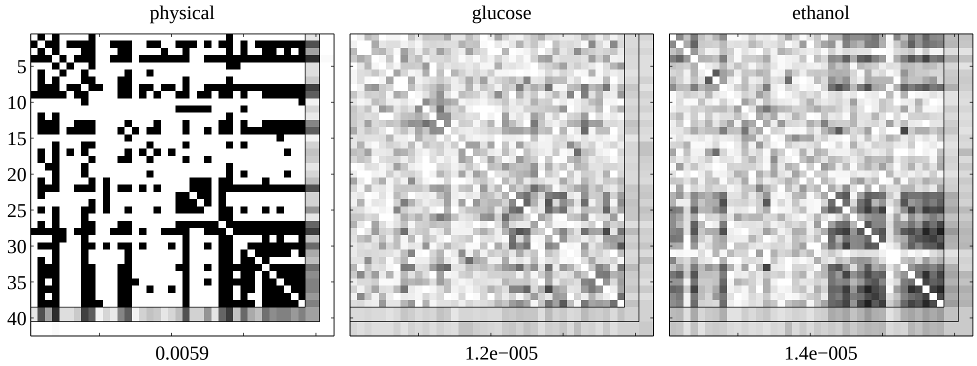

Figure 3. Illustration of a subtle cluster: Cluster #52 in JointCluster's clustering of the yeast physical and coexpression networks. Heatmap conventions are the same as in Figure 1.

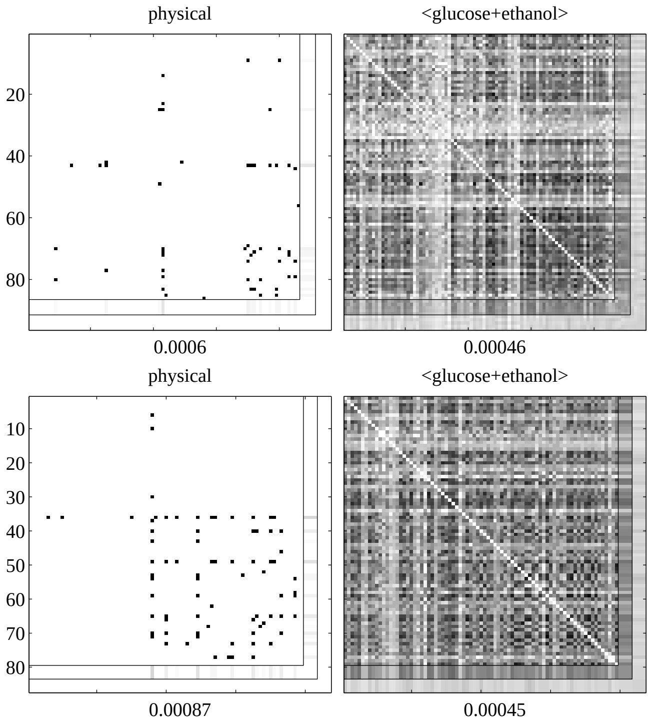

Figure 4. Example clusters identified by JointCluster in a two-network clustering of <glucose+ethanol> and physical networks, despite poor physical connectivity of the clusters. These clusters were significantly enriched for GO processes related to mitochondrion (protein targeting to mitochondrion process with $P = 1.7\text{e-}23$ for the top cluster, and mitochondrion organization with $P = 5.3\text{e-}19$ for the bottom cluster), and were both top-ranked in the two-network clustering. A large fraction of genes in the clusters (64 of 86 genes in the top and 39 of 79 genes in the bottom cluster) were not present in any of the clusters detected by Matisse in the same two-network clustering. Matisse enforces connectivity in the physical network when finding clusters, whereas JointCluster use a more flexible framework in integrating multiple networks. Extreme examples of genes missed by Matisse clusters were genes with no interaction partners in the physical network. Such physically isolated genes in the top/bottom cluster were respectively: {ACN9, POA1, YPL098C, ERV1, YMR166C, YOR366W, YNL140C} and {EMI5, ODC1, MDM35, PET191, YML087C}. Heatmap conventions are the same as in Figure 1, except that the heatmap is based on an ordering of cluster genes that places physically isolated genes first and other genes missed by Matisse clusters next before the remaining genes.

Table 1. Many clusters preserved in the yeast physical and glucose/ethanol coexpression networks were significantly enriched for a GO Process term. Only the enrichment results for the top-25 ranked clusters at a significance level of 0.005 (after Bonferroni correction for the number of reference sets in GO Process class) are shown here, with the rank of a cluster given by an ordering of all clusters in the detected clustering by their min-modularity scores. Enrichment P-value was calculated using the 4482 genes in the networks as the background.

Cluster Rank or Identifier	GO Process Term	Enrichment P-value (P)	# Genes in Cluster	# Genes overlapping cluster and reference set	# Genes in reference set
#1	translation	1e-20	82	38	367
#2	ribosome biogenesis	2.4e-37	76	59	609
#4	rRNA processing	2e-47	85	48	176
#5	Golgi vesicle transport	1.5e-08	95	17	155
#6	chromosome segregation	2.1e-06	90	12	114
#7	protein targeting to mitochondrion	3.6e-17	68	15	46
#8	vacuolar transport	1.2e-07	87	13	109
#12	mitochondrial translation	4.5e-33	72	28	76
#13	amino acid biosynthetic process	1.1e-16	96	20	82
#15	translation	9.1e-41	87	56	367
#16	negative regulation of macromolecule metabolic process	1.9e-06	70	15	233
#19	oxidation reduction	1.6e-25	40	17	44
#20	maturation of 5.8S rRNA	4.4e-07	77	9	58
#21	gene silencing	3.8e-06	81	10	90
#22	ribosome biogenesis	6.2e-07	35	17	609

Table 2. Biologically significant clusters are clusters enriched for some gene set in all five reference classes: GO Process, TF Perturbations, Compendium of Perturbations, TF Binding Sites, and eQTL Hotspots (denoted as I to V respectively in the second column). The biologically significant clusters detected by JointCluster on simultaneously clustering the yeast physical and glucose/ethanol coexpression networks, and the reference sets for which they were enriched is shown. The columns have the same interpretation as Table 1.

Cluster Rank or Identifier	Reference class : reference set	Enrichment P-value (P)	# Genes in Cluster	# Genes overlapping cluster and reference set	# Genes in reference set
#4	I : rRNA processing	2e-47	85	48	176
	II : SNF2 deletion	2.9e-06	85	26	534
	III : BUD22 deletion	1e-10	85	23	252
	IV : ABF1 binding sites	1.1e-05	85	18	305
	V : glu12 hotspot	8.1e-25	85	67	1159
#13	I : amino acid biosynthetic process	1.1e-16	96	20	82
	II : GCN4 overexpression	4e-24	96	31	143
	III : GCN4 deletion	3e-32	96	29	68
	IV : GCN4 binding sites	1.6e-39	96	43	153
	V : eth2 hotspot	3.5e-07	96	6	15
#15	I : translation	9.1e-41	87	56	367
	II : SPT10 deletion	9.4e-28	87	51	492
	III : ERG11 (tet promoter) deletion	1.6e-06	87	45	1244
	IV : FHL1 binding sites	1.2e-32	87	33	109
	V : gxe8 hotspot	1.6e-09	87	14	93
#19	I : oxidation reduction	1.6e-25	40	17	44
	II : SUT1 overexpression	9.5e-16	40	19	206
	III : RTS1 deletion	2.3e-11	40	12	99
	IV : HAP4 binding sites	2.8e-23	40	16	45
	V : gxe11 hotspot	5.5e-20	40	21	175
#28	I : multi-organism process	2.5e-12	50	14	104
	II : STE12 overexpression	5.7e-09	50	11	98
	III : FUS3, KSS1 (haploid) deletion	8.1e-21	50	14	30
	IV : DIG1 binding sites	9.3e-30	50	27	129
	V : glu7 hotspot	2.2e-12	50	11	49
